# Young but not defenceless: antifungal activity during embryonic development of a social insect

**DOI:** 10.1098/rsos.191418

**Published:** 2020-08-26

**Authors:** Erin L. Cole, Haley Bayne, Rebeca B. Rosengaus

**Affiliations:** Department of Marine and Environmental Sciences, Northeastern University, 134 Mugar Building, 360 Huntington Avenue, Boston, MA 02115, USA

**Keywords:** eco-immunology, fungal entomopathogen, mycosis, social immunity, termite, embryogenesis

## Abstract

Termites live in environments heavily colonized by diverse microorganisms, including pathogens. Eggs laid within the nest are likely to experience similar pathogenic pressures as those experienced by older nest-mates. Consequently, eggs may be under selective pressures to be immune-competent. Through *in vitro* experiments using developing embryos of the dampwood termite, *Zootermopsis angusticollis*, we tested the ontogeny, location and strength of their antifungal activity against the fungus, *Metarhizium brunneum*. Exterior washes of the chorion (extra-chorionic) and components within the chorion (intra-chorionic) were incubated with fungal conidia, which were then scored for viability. The fungistatic activity was location and developmental stage dependent. Extra-chorionic washes had relatively weak antifungal activity. Intra-chorionic homogenates were highly antifungal, exhibiting increased potency through development. The positive correlation between intra-chorionic fungistasis and developmental stage is probably due to the expression of endogenous proteins during embryogenesis. Boiling of both the extra-chorionic washes and the intra-chorionic contents rescued conidia viability, indicating the antifungal agent(s) is (are) heat-sensitive and probably proteinaceous. This study is the first to address embryonic antifungal activity in a hemimetabolous, eusocial taxon. Our results support the hypothesis that microbes have been significant agents of selection in termites, fostering the evolution of antifungal properties even in the most immature stage of development.

## 1. Introduction

Insects have effective immune responses against a diverse array of pathogens [1–3]. However, the long-held view that immature developmental stages lack immune-competency and thus have higher susceptibility to infectious pathogens [4–11] is not supported by current evidence. An increasing number of studies demonstrate that insect embryos are capable of mounting immune-defences [12–21]. These studies, however, are heavily biased towards holometabolous, solitary insects. Back in 2002, Ayasse & Paxton [8] claimed that social insect eggs lack chemical defences. Since then, only one study on the holometabolous social bumblebees has addressed and debunked this claim [22]. To the best of our knowledge, no other study has investigated embryonic antimicrobial defences across any other social insect species.

Termite colonies are heavily colonized by diverse microbial communities (reviewed in [23,24]), including multiple fungal pathogens [25–27]. Because pathogens represent strong agents of selection (e.g. [3,28–30]), we hypothesized that natural selection should have favoured the evolution of embryonic defences, particularly in soil- and decayed wood-dwelling species. While a lysozyme was identified in eggs of the subterranean termite, *Reticulitermes speratus* [31], and lysozymes are generally considered antimicrobial in nature (e.g. [32]), this particular egg-associated enzyme was only identified as an egg recognition pheromone [31]. Neither the antimicrobial properties of this lysozyme [31], nor any other embryonic compound(s) of termite eggs, have ever been tested against actual pathogens.

Several, not mutually exclusive, sources of embryonic protection may exist. First, mutualistic microbes colonizing the outer surface of the eggshell (chorion) may produce potent antifungal compounds [33]. For example, actinomycete-derived compounds help fungus-growing ants and bee-wolf wasps protect their fungus garden and progeny, respectively [34,35]. Second, a queen may coat the outer-layer of the chorion with protective secretions as eggs traverse her reproductive tract and/or during oviposition [36,37]. Third, licking of the outer surface of the chorion by parents and nest-mates may result in the physical removal of microbes [38,39] and/or the deposition of salivary gland antifungal compounds, such as lysozymes (e.g. [31,32,40–42]) and β-1,3 glucanases [43–45]. These compounds can deactivate microbes, thereby reducing the risk of infection in eggs. Fourth, the queen may imbue her eggs with prefabricated antifungal compounds during oogenesis such as defensins [46], vitellogenins [47–51] and lysozymes [31,32,40–42]. Lastly, immature termite embryos may be independently immuno-competent as reported in *Tribolium castaneum* and *Tenebrio molitor* [16–18,20]. Regardless of its origin, antifungal activity on, or within, the chorion of termite eggs could have profound impacts on the disease ecology of the colony.

Here, we used changes in conidia viability as a proxy to determine if *Zootermopsis angusticollis* embryos have antifungal properties against the fungus *Metarhizium brunneum*. Given the limited information on embryogenesis in *Z. angusticollis* [52], we first had to visually characterize embryonic developmental stages of *Z. angusticollis.* This staging protocol allowed us to explore the ontogeny of antifungal defences. We subsequently tested the location of the fungistatic properties on the surface of the chorion (extra-chorionic) or within the chorion (intra-chorionic) and the relative strength of the observed fungistatic activity. Our data provide the first evidence of antifungal activity of termite embryos. These results, viewed through the lens of eco-immunology, point to the significant role that fungal pathogens have probably played in the evolution of pathogenic defences across all stages of development in this social insect taxon.

## 2. Material and methods

### 2.1. Characterization of embryonic developmental stages

**Figure 1.**
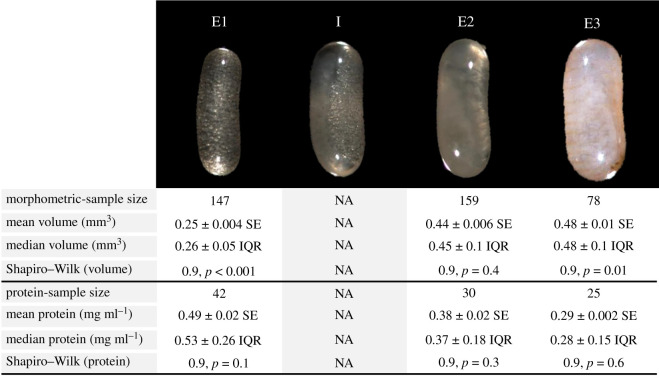
Developmental stages of *Z. angusticollis* embryos and the descriptive statistics for morphometric and total protein content data. ‘E1’ = stage 1 embryos, younger than 7 days and filled with ‘vitellogenic bubbles’. ‘I’ = example of an intermediate stage with both vitellogenic bubbles and initial tissue condensation (opaque whitish tissue on the left side of the embryo). ‘I’ embryos were not used in these experiments. Their volume and protein content are not available (NA). ‘E2’ = stage 2 embryos, opaque tissue with no vitellogenic bubbles. ‘E3’ = stage 3 embryos, fully formed termite with segmentation and defined body parts visible through chorion. SE, standard error; IQR, interquartile range. Statistics and *p*-values are shown for Shapiro–Wilk tests of normality. The contrast and brightness of these images were manipulated to display the specific traits on which our embryonic staging was based.

Surprisingly, termite embryology has received relatively little attention [52]. Since Krishna and Weesner in 1970 [52], the available data on the visual characteristics and timing of termite embryological development came from *R. speratus* [53]. To investigate the ontogeny of antifungal activity in *Z. angusticollis*, we first characterized each embryological stage as a broad approximation of chronological age. The exact chronological age was impossible to pinpoint given that eggs from mature colonies are always laid inside tunnels/chambers deep within the decayed wood. In incipient colonies of *Z. angusticollis*, where timing of oviposition is easier to observe, embryos take approximately 60–70 days from oviposition to hatching [54]. Assuming the developmental timeline is identical in mature colonies, our E1 (the earliest developmental stage, defined in figure 1) from mature colonies were visually consistent with embryos less than one week old, whereas E3 (the most developed stage prior to hatching, figure 1) were visually consistent with embryos in the final days of development before hatching. We, henceforth, refer to the termite eggs as ‘embryos’ to draw a distinction between the haploid female gamete (egg) and the diploid (fertilized and oviposited) developing eggs (embryos). We assumed all oviposited eggs were fertilized.

Embryos were retrieved from seven mature colonies, all collected from the Redwood East Bay Regional Park, in Oakland, California. These colonies were maintained inside closed plastic tubs at our USDA-approved containment room (permit no. P526P-17-03817). Colonies were provided with white birch (*Betula*, as both nesting and feeding material) ad libitum and periodically sprayed with water while kept at 25°C. The embryos were recovered after breaking apart nest material and removing them with featherweight forceps from BioQuip (figure 1 and electronic supplementary material, table S1 for sample sizes/colony). The embryos were then examined under a dissecting microscope at 40× magnification and visually categorized into three developmental stages (figure 1) using a modified protocol from that of Matsuura & Kobayashi [53]. Stage 1 embryos (E1), the most immature stage [52–55], had a characteristic ‘bubbly’ appearance (‘yolk spheres’; [55, p. 512]), presumably the result of high vitellogenin content (storage yolk protein; [48]) visible through the chorion (figure 1). Stage 2 embryos (E2) had opaque tissue condensation with no evidence of vitellogenin bubbles (figure 1). Stage 3 (E3) encompasses a short phase of development just prior to hatching, characterized by amber-coloured abdominal segmentation and other anatomical features visible through the chorion (head, antennae and legs; figure 1). We purposefully avoided using ‘intermediate stage’ embryos (‘I’ in figure 1). The same staging protocol was used to characterize embryos throughout the study. To validate that our visual staging protocol (figure 1) truly represented different developmental stages, we also compared embryonic size (i.e. volume) across the three embryological stages and quantified their overall protein content (see below).

### 2.2. Morphometric analyses of embryonic stages

Volume of individual embryos (sample sizes in figure 1) was estimated by photographing each embryo (at 40× magnification; Qspot camera) and measuring its length and width using SPOT imaging software. Embryo volume was estimated with the formula:

$$V=\pi {\left(\frac{W}{2}\right)}^{2}L,$$

where *W* = width and *L* = length [56].

### 2.3. Protein concentration

Quantifying protein content of embryos served two important purposes: (i) validate that our visual categorization represented different physiological states (figure 1) and (ii) account for possible effects of protein concentration on embryonic antifungal properties, whether extra- or intra-chorionic (see below). We quantified total protein content of the samples using the Bio-Rad Protein Assay. Although measuring protein content of single embryos would have been ideal, due to sensitivity limitations of this assay, we were forced to pool three same stage and same colony of origin (COO) embryos into a single sample. After imaging, these three same-stage/COO embryos were transferred into a 1.6 ml sterile microcentrifuge tube with 200 µl of sterile 1× phosphate-buffered saline (PBS) with 1× Pierce™ EDTA-free protease inhibitor cocktail (PI). These samples were sonicated (Vibra-Cell™ Model: CU18) while on ice at an amplitude of 35% for three 5 s pulses. For each sample, we visually confirmed that the chorion had ruptured and that the solution became opaque (i.e. ‘milky’) after sonication. Following sonication, the sample was then centrifuged gently to pull down any larger remnants of the chorion. The supernatant was then transferred to a fresh microcentrifuge tube and immediately flash frozen in liquid nitrogen, then stored at −80°C. Prior to protein quantification, the samples were thawed and thoroughly vortexed. We followed the procedural instructions accompanying the kit, using a 96-well plate and read at 595 nm (BioTek plate reader with Gen5 Data Analysis Software). The protein content of each sample was measured in duplicate, and all statistical analyses were conducted on the average of the two reads/sample. For the extra-chorionic washes, the protein assay was only carried out on the supernatant following the wash protocol (see below). For the intra-chorionic homogenates, the protein assay was carried out on the sonicated sample following the wash protocol (see below).

### 2.4. Preparation of fungal conidia suspensions

*Metarhizium* is a common and geographically widespread entomopathogenic soil fungus known to infect termites and their nests (e.g. [27,57–60]). Conidia show a high affinity to the chorion, binding to and germinating on the outer surface of *Zootermopsis* embryos (electronic supplementary material, figure S1). Therefore, this fungus represents an ecologically relevant pathogen. Notably, fungal conidia are the actual infectious propagule that results in insect mycosis [27,57–60]. Hence, we tested termite embryos against conidia in order to approximate natural infection scenarios.

Our strain of *Metarhizium* was purchased from American Type Culture Collection (batch no. 9309, media no. 325, ATCC 90448, originally sold as *Metarhizium anisopliae*). This strain has since been re-classified by ATCC as *M. brunneum* [61]. Cultures of *M. brunneum* were grown by first infecting *Zootermopsis* nymphs with the infectious conidia and allowing the infectious process to continue until the nymphs died. The cadavers were then surface-sterilized in 6% sodium hypochlorite, followed by two sterile water washes and plated onto potato dextrose agar (PDA) until sporulation [62,63]. These conidia were subsequently harvested and transferred into a sterile 0.1% Tween 80 suspension [63]. This method ensured that all of our embryonic experiments were carried out with fresh, viable and highly virulent (germination rates ranging between 90 and 94%) conidia stock.

### 2.5. Antifungal activity

The following conidia viability assays allowed us to evaluate embryonic antifungal activity in two different locations: extra-chorionic and intra-chorionic, and across three different embryological stages (E1–E3). Such assays are commonly used to detect fungistatic properties (e.g. [62–66]), and in this case, tested whether the viability of the natural infectious propagules (i.e. conidia) are affected after incubation with embryonic washes and homogenates of embryonic tissues.

### 2.6. Experiment 1: extra-chorionic antifungal activity

**Figure 2.**
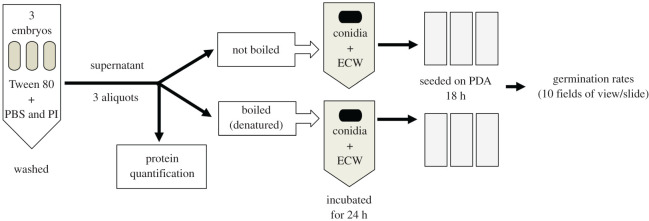
Experimental design for the extra-chorionic wash antifungal assay. Light ovals, embryos; ECW, extra-chorionic wash; black ovals, conidia of *M. brunneum*.

A total of 231 embryos from seven mature colonies (different from those used in the characterization of embryological stages, see above) were collected and sorted into 77 different samples, each containing three embryos of the same stage and same COO (for sample sizes, see Results and electronic supplementary material, table S2). Immediately following collection, staging (figure 1), imaging and sorting, embryos were then submerged in 200 µl of a PBS with 1× PI solution and shaken by hand for 1 min (figure 2). Subsequently, 5 µl of a 0.1% Tween 80 solution (a surfactant) was added to the mixture, then vigorously shaken by hand for 30 s to foster detachment of putative microbes and other possible compounds resting on the chorion's outer surface. Once the intact embryos sank to the bottom of the microcentrifuge tube, the supernatant (i.e. extra-chorionic wash) was divided into three aliquots (figure 2): 20 µl were used to quantify total soluble protein (see protocols above), 60 µl were boiled in a water bath for two minutes to denature putative proteins and 60 µl remained unmanipulated. The second and third aliquots were each incubated with 60 µl of a 1 × 10^7^ conidia ml^−1^ stock suspension for 24 h at 25°C on a rotating plate (150 r.p.m.). Given that a 1 × 10^7^ conidia ml^−1^ suspension is a relatively high and lethal concentration [67], a reduction of conidia germination at this high load would be a robust indicator of antifungal properties. Following incubation, 10 µl of the mixture (containing both conidia and chorion surface wash) were seeded onto a microscope slide containing 1 ml of solidified PDA (see [58,59] for details). Each sample was run in triplicate (i.e. three slides per sample, figure 2). These seeded slides were immediately inspected to determine if conidia had germinated during the 24 h incubation period. No conidia germinated during the incubation with the extra-chorionic washes. The seeded slides were cultured for 18 h at 25°C inside a covered plastic box lined with moist paper towel to maintain humidity at roughly 98%. Counting conidia germination at 18 h post-seeding is optimal as these infectious propagules start growing their germ tube after approximately 12–16 h (RB Rosengaus 1998, personal observation). After 18 h, the germ tubes cannot be accurately counted. These seeded slides were then viewed at 400× magnification to count the proportion of conidia with visible germ tubes (i.e. per cent germination, a measure of conidia viability; [62–66]). The viability of conidia germinated in the PBS/PI/Tween 80 cocktail did not differ from conidia suspended in Tween 80 alone (Mann–Whitney *U* test: *z* = −1.2, *p* = 0.2). Conidia germinated in PBS/PI/Tween 80 solution were used as the control treatment in subsequent statistical analyses.

### 2.7. Experiment 2: intra-chorionic antifungal activity

**Figure 3.**
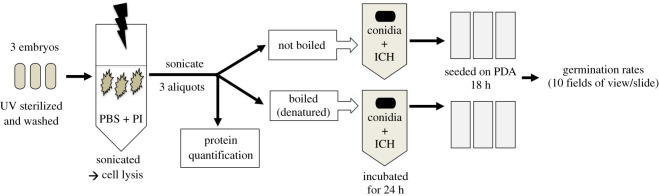
Following UV irradiation, embryos underwent the depicted experimental design to assess intra-chorionic fungistatic activity. Light ovals, embryos; black lightning bolt, sonication; 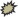, lysed embryos, ICH, intra-chorionic homogenate (i.e. the sonicate), black ovals, conidia of *M. brunneum*.

To test whether fungistatic activity originated from within the chorion, embryos from the same seven mature colonies used in Experiment 1 were collected and sorted into 87 different samples (three same-stage, same-colony embryos pooled per sample; see Results and electronic supplementary material, table S3 for sample sizes). Immediately following collection, staging and imaging, we surface-sterilized the outer chorion of intact embryos to reduce any putative sources of antimicrobial factors that could have affected our results. To this end, embryos were placed in a glass Petri dish and exposed to UV light (200 µW cm^−2^) for a total of 2 min, shaking the dish every 30 s to ensure the entire embryonic surface area was irradiated (figure 3). This protocol reduced, but did not eliminate, the microbial load of the outer surface of the chorion from an average combined fungal and bacterial load of 8.2 ± 5.4 to 2.9 ± 2.0 CFUs/50 µl (electronic supplementary material, Methods and Data S1). Following UV irradiation, the embryos were washed as described above and then sonicated in 200 µl of fresh and sterile PBS/PI/Tween 80 cocktail and divided into three aliquots (figure 3): 20 µl were used to quantify total soluble protein (see above), 60 µl were boiled in a water bath for 2 min to denature putative proteins and 60 µl remained unmanipulated. The second and third aliquots were each incubated with 60 µl of a 1 × 10^7^ conidia ml^−1^ stock suspension for 24 h at 25°C on a rotating plate at 150 r.p.m. (figure 3). Per cent germination was determined exactly in the same fashion as for the extra-chorionic protocols described earlier (figure 3).

### 2.8. Statistical analyses

All statistical analyses were carried out using IBM SPSS, v. 25.

### 2.9. Embryonic volume and protein quantification

Embryonic volume was not normally distributed across embryonic stages (figure 1). We built a general linear mixed effect model (LMM) fitted to a normal distribution to test the independent effect of embryonic stage (fixed effect) on volume while controlling for the COO (random effect), followed by *post hoc* multiple comparisons (adjusting with a Bonferroni correction). Because the model residuals were normally distributed, this model was appropriate for testing our hypothesis that embryo volume differed by embryonic stage.

Protein content was normally distributed across embryonic stages (figure 1). Total protein content was analysed via LMM, fitted to a normal distribution, as a function of embryonic stage (fixed effect), total volume of the three pooled embryos (covariate) and COO (random effect). Pairwise *post hoc* multiple comparisons were performed (Bonferroni correction).

### 2.10. Antifungal activity

Percentages are inherently not normally distributed, hence we used non-parametric methods to test our hypothesis that the germination rates of conidia incubated with embryo washes or homogenates would differ from those of conidia incubated without embryonic washes or homogenates (controls). Given that the extra- and intra-chorionic protocols differed from one another, we ran separate Kruskal–Wallis (KW) tests for each experiment, with germination rate as the response variable. The explanatory variable had seven levels: controls, E1 boiled and unboiled, E2 boiled and unboiled and E3 boiled and unboiled. *Post hoc* tests (Mann–Whitney; MW) for all possible pairwise comparisons were performed (Bonferroni correction). Because these KW tests could not control for COO effects nor for differences in volume and protein content, we also generated a separate generalized linear mixed effects model (GLMM) fitted to a binomial distribution (using a binary logit function) for each the extra-chorionic and intra-chorionic experiments. Each individual conidia was scored as either 1 (germinated) or 0 (ungerminated). This dummy variable became the dependent variable. Treatment (i.e. six different categories consisting of each embryonic stage and whether the samples were boiled or unboiled) was included as a fixed effect, while controlling for COO (random effect). Sample ID was included as a random effect to account for repeated measures. Total protein was included as a covariate. Because total protein and total embryo volume were positively correlated, volume was excluded from the model. Control samples inherently lacked embryonic samples or COO information; thus, they were omitted from the GLMM analyses. Hence, we present both the KW tests (which compare samples incubated with and without extra-chorionic washes or intra-chorionic homogenates relative to the control germination rates) and the GLMM (which compare germination rates of conidia only when grown with different embryonic stages while still controlling for COO and total protein).

## 3. Results

### 3.1. Morphometric and protein quantification of embryonic stages

All three embryonic stages were visually distinct (figure 1). After controlling for the effects of COO via LMM, embryonic stage was a significant and independent predictor of embryonic volume (*F* = 685, d.f. = 2, 377.5, *p* < 0.001; figure 1). *Post hoc* multiple pairwise comparisons revealed embryonic volumes between stages differed significantly from each other (E1 versus E2: *T* = 32.5, d.f. = 377.5, *p* < 0.001; E1 versus E3: *T* = 31.1, d.f. = 377.5, *p* < 0.001; E2 versus E3: *T* = 6.8, d.f. = 377.5, *p* < 0.001; figure 1).

Total soluble protein content declined with embryonic development (figure 1). After controlling for the effects of both COO and embryonic volume, embryonic stage was a significant predictor of total protein (*F* = 20.2, d.f. = 2, 17.9, *p* < 0.001). Each stage differed from the others in its total protein content (E1 versus E2: *T* = 4.9, d.f. = 17.9, *p* < 0.001; E1 versus E3: *T* = 6.4, d.f. = 17.9, *p* < 0.001; E2 versus E3: *T* = 3.6, d.f. = 17.9, *p* = 0.002; figure 1). Taken together, data on embryonic volume and protein content validated that the visual staging protocol used in subsequent antifungal experiments accurately reflected physiologically different embryonic stages.

### 3.2. Antifungal properties of termite embryos

**Figure 4.**
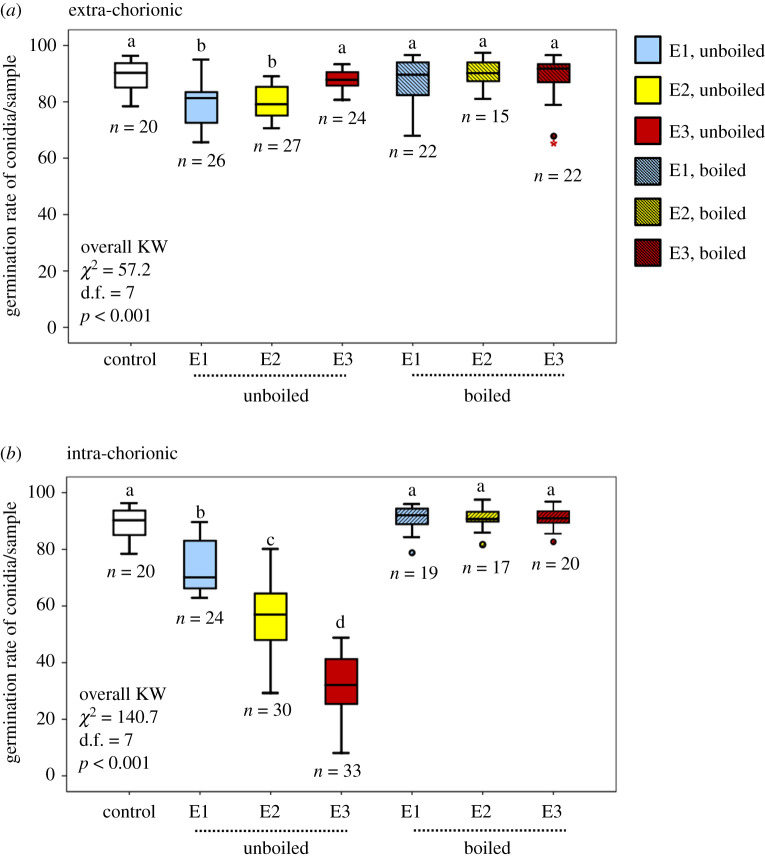
Embryo fungistasis. (*a*) Germination rates of conidia incubated with unboiled and boiled washes of the chorion's exterior surface. (*b*) Germinations rates of conidia incubated with unboiled and boiled contents from within the chorion. Different letters denote statistical significance in pairwise comparisons following a Bonferroni correction (see electronic supplementary material, tables S4 and S6). *n* = number of samples (each consisting of three same-stage embryos from the same COO). ° and * represent outliers and extreme outliers, respectively.

Our results indicate embryos have two independent locations of fungistatic activity (figure 4*a* and *b*). The first, located on the outer surface of the chorion, provides a relatively weak protection for stages E1 and E2 (figure 4*a*). The second, significantly more potent than the extra-chorionic fungistasis, is located within the chorion. The data from each experiment are described in detail below.

### 3.3. Experiment 1: extra-chorionic antifungal activity

Overall, conidia germination after incubation with extra-chorionic washes was significantly lower than that of control conidia (KW: *χ*^2^ = 57.2, d.f. = 7, *p* < 0.001; figure 4*a*). However, the *post hoc* Mann–Whitney U tests revealed that only E1 and E2 stages differed from control conidia (*U* = 92, d.f. = 1, *p* < 0.001; *U* = 93, d.f. = 1, *p* < 0.001, respectively). Conidia incubated with E3 extra-chorionic washes as well as conidia incubated with boiled washes, irrespective of embryonic stage, did not differ from control conidia (figure 4*a*; electronic supplementary material, table S4). After controlling for both COO (*Z* = 1.1, d.f. = 13 488, *p* = 0.3) and total protein concentration (*F* = 0.3, d.f. = 1, 13 488, *p* = 0.6), extra-chorionic washes had a marginally significant fungistatic effect on *Metarhizium* conidia (*F* = 2.1, d.f. = 11, 13 488, *p* = 0.06, GLMM). E1 extra-chorionic washes significantly decreased conidia viability relative to all other stages. The antifungal properties of E2 and E3 washes did not differ significantly from each other. When conidia were incubated with boiled extra-chorionic washes, the fungistatic nature of such washes was lost. This was true regardless of embryonic stage (figure 4*a*; electronic supplementary material, table S5). The interaction treatment × total protein was not significant (*F* = 1.7, d.f. = 5, 13 488, *p* = 0.1).

### 3.4. Experiment 2: intra-chorionic antifungal activity

Conidia incubated with unboiled intra-chorionic homogenates of any stage had significantly lower germination rates than control conidia and boiled intra-chorionic homogenates (figure 4*b*; electronic supplementary material, table S6). Boiled samples did not significantly differ from controls, indicating that boiling ‘rescued’ the conidia (figure 4*b*; electronic supplementary material, table S6). The GLMM showed the same pattern. After controlling for COO (*Z* = 0.9, d.f. = 14 288, *p* = 0.4) and total protein (*F* = 0.2, d.f. = 1, 14 288, *p* = 0.6), embryonic stage was a significant predictor of conidia germination rates (*F* = 83.2, d.f. = 5, 14 288, *p* < 0.001). All three embryonic stages differed significantly from each other with increasing antifungal activity as embryos progressed through development (figure 4*b*; electronic supplementary material, table S7). Although the interaction treatment × total protein was significant (*F* = 2.7, d.f. = 5, 14 288, *p* = 0.02), this significance appears to have been driven by the total protein outliers; none of the *post hoc* comparisons were significantly different from each other. Graphs of these interactions also support the conclusion that total protein explains only a small amount of the variation observed between treatments (with *R*^2^ estimates ranging from 0.02 to 0.25; electronic supplementary material, figure S2).

## 4. Discussion

Our experimental design approximated the usual sequence of events that culminate in infection. In nature, fungal conidia (the infective propagules of this fungus) first first adhere to (via the formation of appressoria), and then germinate on the insect’s exoskeleton [68]. These two first steps in the infectious process also occur over the exterior surface of termite embryos (electronic supplementary material, figure S1), indicating that the embryonic chorion is a suitable substrate for *Metarhizium* conidia. The embryo-phillic tendencies of conidia observed in the laboratory (electronic supplementary material, figure S1) suggest that embryonic infection is likely to occur under natural conditions as well.

Termite embryos exhibited antifungal activity against the entomopathogenic fungus *M. brunneum* (figure 4*a,b*). While extra-chorionic protection is relatively weak and short-lived (present only in E1 and E2, reducing conidia viability by approximately 10–12% relative to controls; figure 4*a*), intra-chorionic protection is potent and positively correlated with developmental stage (with E1 reducing germination by approximately 20% and E3 by approximately 60%; figure 4*b*). These patterns remained significant even after controlling for the effect of both COO and total protein (electronic supplementary material, tables S5 and S7). Conidia germination was rescued when extra-chorionic washes and intra-chorionic homogenates were boiled (figure 4*a,b*, respectively), demonstrating that the antifungal agent(s) is (are) sensitive to heat and thus, likely to be one or more protein(s).

The present work helps reveal some of the likely source(s) of the presumed antifungal compounds. Extra-chorionic fungistasis, for example, could have resulted from either microbial by-products, the deposition of salivary gland secretions during grooming by parents and nest-mates, and/or maternal coating/smearing during oviposition. Embryos receive intense brood care from kings, queens and workers [69–71]. Such behaviours are likely to result in the physical removal of microbes from the surface of the chorion, a strategy akin to that reported during mutual grooming among workers [38]. Embryo grooming/licking may also result in the deposition of salivary gland secretions on the chorion's exterior, which are known to possess antimicrobial properties (e.g. [44,45,71]). Specifically, active β-1,3-glucanases are excreted by the termite's salivary glands and are known to break down fungal cell walls [44]. Further support for the role of salivary gland secretions containing antifungal compounds comes from the fact that E1 extra-chorionic washes had 2.5 mg ml^−1^ more protein than extra-chorionic washes of E2 and E3 (electronic supplementary material, Data S2). Hence, the possibility exists that E1 are preferentially groomed/licked by parents/workers, and/or that the efficacy of the salivary antimicrobial compound(s) is temporary, waning as E1 embryos progress through their development. Currently, no behavioural data exist on whether nest-mates or reproductives preferentially groom/lick younger embryos over older ones. Unfortunately, our extra-chorionic experiments could not conclusively distinguish among the three possible sources of fungistasis (microbial by-products, salivary gland secretions or coating/smearing during oviposition). The reality is that all three sources, or any combination thereof, could play a role in the external protection of embryos.

The intra-chorionic experiments allow for more precise inferences regarding the source of fungistasis. The reduction in germination seen in conidia incubated with E1 relative to control conidia may originate from prefabricated compounds that queens imbue into their embryos, such as vitellogenins [31]. This yolk precursor is known to have antimicrobial activity and plays a role in immunity across a variety of taxonomical groups (e.g. [32,40–42,47,49–51]). Our results indicate that E1 embryos, which probably have the highest concentration of maternally contributed vitellogenins before becoming metabolized during embryogenesis, reduced conidia germination by approximately 10% (figure 4*b*). Given E1's significant difference in antifungal properties relative to the controls, we posit that queens may convey slight protection to their embryos via their vitellogenins. These maternally contributed compounds may be beneficial to the developing embryo before the expression of the embryo's endogenous antifungal compounds begins. Vitellogenins, however, do not appear to be the main and/or most potent antifungal compound(s) within the embryos since the vitellogenin-rich E1 had the weakest intra-chorionic fungistasis (figure 4*b*), while E3 (with no obvious vitellogenin bubbles; figure 1) exhibited the highest fungistasis (figure 4*b*). We doubt that microbial by-products within the chorion played a significant role in the fungistasis. Otherwise, we would have expected a relatively uniform level of fungistasis across all three embryonic stages, a pattern that was not observed. Instead, our results, using experimentally naive embryos from mature colonies, strongly suggests that the intra-chorionic antifungal activity results from constitutive (as defined by Boots and Best; [72]), endogenous defences. The positive correlation between intra-chorionic fungistasis and developmental stage (figure 4*b*) provides strong circumstantial evidence that stage-dependent intra-chorionic antifungal activity is derived from the embryo itself during development. Given that pathogens are ubiquitous and represent significant agents of selection [28–30], it is reasonable to expect the evolution of effective defences, even in the most immature and supposedly most vulnerable stage of development.

Natural selection could have selected for different strategies that ultimately rendered termite embryos less susceptible to mycosis. Eggs could have been selected to have higher fungistasis on the outer surface of the chorion as a first line of defence, a strategy that would ultimately benefit the developing embryo by reducing the initial stages of fungal invasion. Alternatively, natural selection could have fostered significant investment in antifungal properties within the chorion at the expense of external protection. This scenario is particularly plausible when considering the social nature of these insects and the intense egg-licking behaviour of parents and workers. Grooming of eggs could have ‘emancipated’ the developing embryo from investing in external protection and, instead, invest in within-chorion immune-defences as a secondary layer of protection. Notably, there is no reason why external and internal embryonic adaptations to reduce mycosis should be pitted against each other. Extra- and intra-chorionic protection should not be considered as either/or alternatives against disease, but rather complementary to each other. Our results provide a strong foundation for future comparative studies into the role that developmentally immature stages may play in the facilitation of disease resistance within social insect colonies.

## 5. Conclusion

Our findings have broad evolutionary and eco-immunology implications. We show that termite embryos, although immature, are not defenceless. The combined effects of relatively weak and transient extra-chorionic protection, together with potent and incremental constitutive intra-chorionic defences, are likely to influence the embryos' survival and, consequently, the fitness of the entire colony. This dual protection may result from the dynamic interactions among embryonic microbiomes, social immune benefits from embryonic grooming/licking by nest-mates and the endogenous production of antifungal peptides as embryos develop.

## Supplementary Material

Young but not defenseless: Supplemental Material;Individual Embryo volume;Embryonic total protein;Mycosis Master File;Mycosis Conidia Scores

Reviewer comments

## Data accessibility

Our data have been deposited with RSOS as four electronic supplementary material, excel files as follows: ‘Egg volume’ contains the morphometric measurements and calculated volumes of individual embryos. ‘Total Protein’ contains the protein assay data of each sample (containing three pooled eggs) and their combined morphometric data. ‘Mycosis_masterfile’ contains all relevant data from antifungal experiments 1 and 2, coded as per cent germination. ‘Mycosis_conidia_scores' contains all relevant data from antifungal experiments 1 and 2, with each conidia coded as germinated or ungerminated. All files are annotated with comments.
