# Supplementary material for: Young but not defenceless: antifungal activity during embryonic development of a social insect
Source: R Soc Open Sci. 2020 Aug 26;7(8):191418. doi: 10.1098/rsos.191418 (PMC7481685; doi:10.1098/rsos.191418)
Supplement: Young but not defenseless: Supplemental Material;Individual Embryo volume;Embryonic total protein;Mycosis Master File;Mycosis Conidia Scores [file rsos191418supp1.docx]

**SUPPLEMENTAL MATERIAL**

**Young but not defenseless: Antifungal activity during embryonic development of a social insect**

Erin L. Cole^*^, Haley Bayne^*^, Rebeca B. Rosengaus^1^

Northeastern University, Department of Marine and Environmental Sciences, 134 Mugar Building, 360 Huntington Avenue, Boston MA 02115. 617-3737032.

cole.eri@husky.neu.edu, bayne.h@husky.neu.edu, r.rosengaus@northeastern.edu

^*^ The authors contributed in equal part to this work.

^1^ To whom correspondence should be addressed

**Supplemental Materials**

**Supplemental Table 1: Sample sizes for the characterization of embryonic stages by COO.**

| **Colony ID:** | **1** | **2** | **3** | **4** | **5** | **6** | **7** | **Total** |
| --- | --- | --- | --- | --- | --- | --- | --- | --- |
| **Stage** |  |  |  |  |  |  |  |  |
| **E1** | 45 | 48 | 15 | 9 | 0 | 24 | 6 | 147 |
| **E2** | 54 | 27 | 15 | 9 | 0 | 48 | 6 | 159 |
| **E3** | 3 | 12 | 15 | 9 | 6 | 27 | 6 | 78 |
| **Total** | 102 | 87 | 45 | 27 | 6 | 99 | 18 | 384 |

Embryonic stages, E1, E2 and E3 described in Figure 1 of the main text.


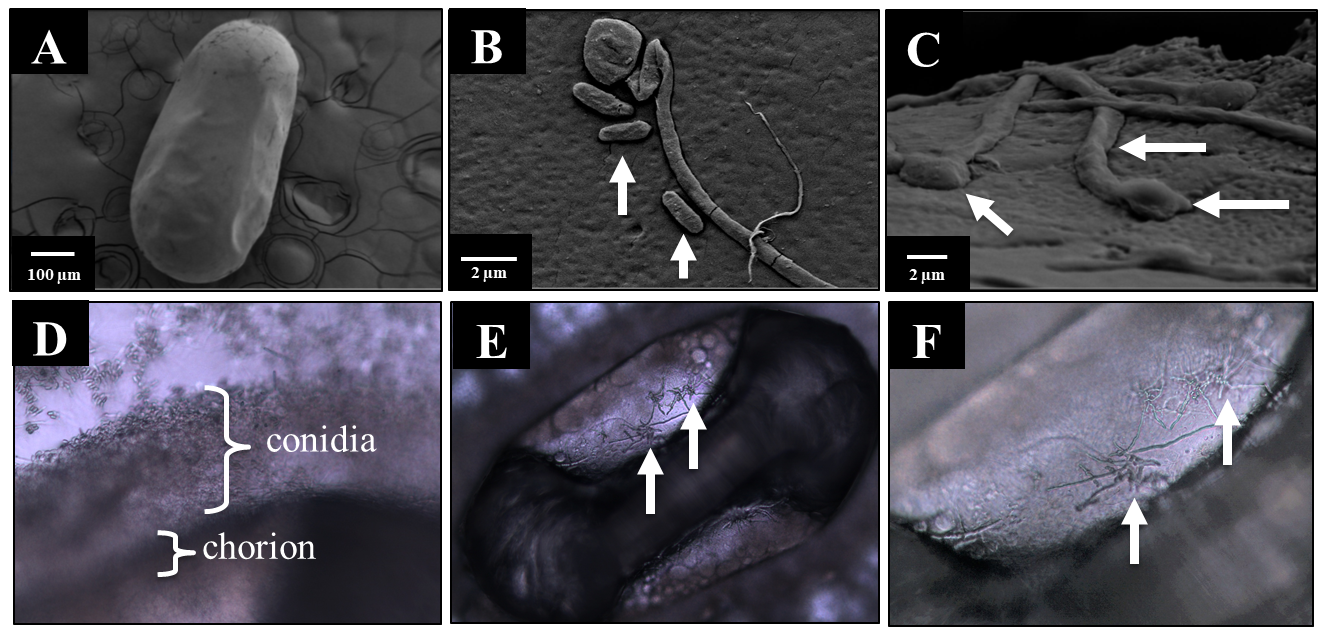


**Supplemental Figure 1: Scanning electron microscopy of an unmanipulated embryo** taken from a mature termite colony (**A – C;** photographed by Dr. Erica Homan, Erica Paige and Catalina Butler at Harvard University) and light microscopy images of embryos experimentally exposed to fungal conidia (**D – F,** photographed by Jeremy McDavid at Northeastern University). **A)** Whole embryo imaged via SEM. **B)** Surface of the embryo with unidentified microorganisms (presumably fungi) adhering to the chorion’s exterior surface (white arrows). **C)** A different section of same embryo (B), with white arrows pointing to what appears to be germination tubes of an unidentified fungus. **D)** Heavy aggregation of *M. brunneum* conidia at the boundary of the chorion (400X magnification) and the surrounding environment after the embryo was submerged in conidia suspension. **E)** Entire embryo (100X magnification) with white arrows pointing to germination tubes of *M. brunneum*. **F)** Same as E but at 400X magnification, with arrows pointing to the same two clusters of germinated conidia.

**Samples sizes for antifungal experiments by COO and embryonic stage**

**Supplemental Table 2: Samples sizes for extra-chorionic experiment.**

| **Colony ID:** | **2** | **8** | **9** | **10** | **11** | **12** | **13** | **Total** |
| --- | --- | --- | --- | --- | --- | --- | --- | --- |
| **Stage:** |  |  |  |  |  |  |  |  |
| **E1** | 2 | 6 | 3 | 2 | 7 | 6 | 0 | 26 |
| **E2** | 5 | 5 | 4 | 1 | 0 | 12 | 0 | 27 |
| **E3** | 3 | 3 | 8 | 4 | 1 | 2 | 3 | 24 |
| **Total** | 10 | 14 | 15 | 7 | 8 | 20 | 3 | 77 |

Each sample consists of three embryos. Embryonic stages, E1, E2 and E3 described in Figure 1 of main text.

**Supplemental Table 3: Samples sizes for intra-chorionic experiment.**

| **Colony ID:** | **2** | **8** | **9** | **10** | **11** | **12** | **13** | **Total** |
| --- | --- | --- | --- | --- | --- | --- | --- | --- |
| **Stage:** |  |  |  |  |  |  |  |  |
| **E1** | 2 | 6 | 3 | 2 | 7 | 4 | 0 | 24 |
| **E2** | 5 | 4 | 3 | 1 | 0 | 17 | 0 | 30 |
| **E3** | 2 | 4 | 9 | 5 | 1 | 2 | 10 | 33 |
| **Total** | 9 | 14 | 15 | 8 | 8 | 23 | 10 | 87 |

Each sample consists of 3 embryos. Embryonic stages, E1, E2 and E3 described in Figure 1 of the main text.

**Supplementary Methods and Data 1: Effectiveness of Embryo Surface Sterilization.**

***Methods:*** To verify that UV irradiation reduced the microbial community colonizing the outer surface of the chorion, we first estimated the microbial load of non-sterilized (non-irradiated) embryos by washing three same-stage embryos/colony (originating from five different mature colonies) in 200 µL of PBS + PI, along with 5 µL of Tween 80. After vigorous shaking by hand for 30 seconds, 50 µL of the supernatant were plated (in duplicate) onto tryptic soy agar (TSA, fostering bacterial growth) and potato dextrose agar plates (PDA, fostering fungal growth). The Petri dishes were kept at 25 °C for four days in a container lined with a wet paper towel to maintain high humidity. Bacterial and fungal colony forming units (CFUs) were enumerated. A second set of samples were surfaced sterilized by placing three same stage/colony embryos in a glass Petri dish and exposing them to UV light at 200 µW/cm^2^ for a total of two minutes. These surface sterilized embryos were once again washed by placing them in sterile microcentrifuge tubes containing 200 µL of PBS + PI and 5 µL Tween 80, shaken, plated and checked for CFUs as described above.

***Results:*** While the average combined fungal and bacterial CFUs before surface sterilization was 8.2 ± 5.4 SE, the average CFUs recovered from surface-sterilized embryos was 2.9 ± 2.0 SE. Hence, although UV irradiation did not completely eradicate the microbial community colonizing the outer surface of the chorion, it considerably reduced the microbial loads.

**Supplemental Table 4: Pairwise comparisons of antifungal activity for extra-chorionic washes (Mann-Whitney U tests).**

| **Comparison** | | **MWU statistic** | **df** | **p-value** |
| --- | --- | --- | --- | --- |
| **Control** | **Unboiled E1** | **92.0** | **1** | **< 0.001** |
| **Control** | **Unboiled E2** | **93.0** | **1** | **< 0.001** |
| Control | Unboiled E3 | 191.0 | 1 | 0.2 |
| Control | Boiled E1 | 218.5 | 1 | 0.9 |
| Control | Boiled E2 | 129.0 | 1 | 0.5 |
| Control | Boiled E3 | 201.0 | 1 | 0.6 |
| Unboiled E1 | Unboiled E2 | 304.5 | 1 | 0.4 |
| **Unboiled E1** | **Unboiled E3** | **67.0** | **1** | **< 0.001** |
| **Unboiled E1** | **Boiled E1** | **137.5** | **1** | **0.002** |
| **Unboiled E1** | **Boiled E2** | **35.5** | **1** | **< 0.001** |
| **Unboiled E1** | **Boiled E3** | **88.0** | **1** | **< 0.001** |
| **Unboiled E2** | **Unboiled E3** | **93.5** | **1** | **< 0.001** |
| Unboiled E2 | Boiled E1 | 156.0 | 1 | 0.005 |
| **Unboiled E2** | **Boiled E2** | **40.0** | **1** | **< 0.001** |
| **Unboiled E2** | **Boiled E3** | **90.0** | **1** | **< 0.001** |
| Unboiled E3 | Boiled E1 | 247.0 | 1 | 0.7 |
| Unboiled E3 | Boiled E2 | 118.5 | 1 | 0.1 |
| Unboiled E3 | Boiled E3 | 177.0 | 1 | 0.06 |
| Boiled E1 | Boiled E2 | 135.0 | 1 | 0.4 |
| Boiled E1 | Boiled E3 | 220.5 | 1 | 0.6 |
| Boiled E2 | Boiled E3 | 161.5 | 1 | 0.9 |

Bolded rows indicate significance at p ≤ 0.002 following a Bonferroni correction. This table corresponds to letters denoting significance in Figure 4A of the main text.

**Supplemental Table 5: Pairwise comparisons of percent conidia germination following a Generalized linear mixed effects model for extra-chorionic wash experiment.**

| **Comparison** | | **T value** | **df** | **p-value** |
| --- | --- | --- | --- | --- |
| Unboiled E1 | Unboiled E2 | 1.4 | 13488 | 0.2 |
| Unboiled E1 | Unboiled E3 | 2.2 | 13488 | 0.03 |
| **Unboiled E1** | **Boiled E1** | **3.0** | **13488** | **0.003** |
| Unboiled E1 | Boiled E2 | 0.7 | 13488 | 0.5 |
| Unboiled E1 | Boiled E3 | 1.6 | 13488 | 0.1 |
| Unboiled E2 | Unboiled E3 | 1.2 | 13488 | 0.2 |
| Unboiled E2 | Boiled E1 | 1.5 | 13488 | 0.1 |
| Unboiled E2 | Boiled E2 | 0.2 | 13488 | 0.9 |
| Unboiled E2 | Boiled E3 | 0.1 | 13488 | 0.9 |
| Unboiled E3 | Boiled E1 | 0.3 | 13488 | 0.8 |
| Unboiled E3 | Boiled E2 | 0.3 | 13488 | 0.8 |
| Unboiled E3 | Boiled E3 | 1.3 | 13488 | 0.2 |
| Boiled E1 | Boiled E2 | 0.4 | 13488 | 0.7 |
| Boiled E1 | Boiled E3 | 1.5 | 13488 | 0.1 |
| Boiled E2 | Boiled E3 | 0.2 | 13488 | 0.9 |

Bolded rows indicate significance at p ≤ 0.003 following a Bonferroni correction.

**Supplemental Table 6: Pairwise comparisons of antifungal activity for intra-chorionic homogenates (Mann-Whitney U tests).**

| **Comparison** | | **MWU statistic** | **df** | **p-value** |
| --- | --- | --- | --- | --- |
| **Control** | **Unboiled E1** | **57.5** | **1** | **< 0.001** |
| **Control** | **Unboiled E2** | **15.0** | **1** | **< 0.001** |
| **Control** | **Unboiled E3** | **0.0** | **1** | **< 0.001** |
| Control | Boiled E1 | 133.5 | 1 | 0.1 |
| Control | Boiled E2 | 151.0 | 1 | 0.6 |
| Control | Boiled E3 | 163.5 | 1 | 0.3 |
| **Unboiled E1** | **Unboiled E2** | **63.5** | **1** | **< 0.001** |
| **Unboiled E1** | **Unboiled E3** | **0.0** | **1** | **< 0.001** |
| **Unboiled E1** | **Boiled E1** | **17.0** | **1** | **< 0.001** |
| **Unboiled E1** | **Boiled E2** | **20.0** | **1** | **< 0.001** |
| **Unboiled E1** | **Boiled E3** | **13.0** | **1** | **< 0.001** |
| **Unboiled E2** | **Unboiled E3** | **77.0** | **1** | **< 0.001** |
| **Unboiled E2** | **Boiled E1** | **1.0** | **1** | **< 0.001** |
| **Unboiled E2** | **Boiled E2** | **0.0** | **1** | **< 0.001** |
| **Unboiled E2** | **Boiled E3** | **0.0** | **1** | **< 0.001** |
| **Unboiled E3** | **Boiled E1** | **0.0** | **1** | **< 0.001** |
| **Unboiled E3** | **Boiled E2** | **0.0** | **1** | **< 0.001** |
| **Unboiled E3** | **Boiled E3** | **0.0** | **1** | **< 0.001** |
| Boiled E1 | Boiled E2 | 129.0 | 1 | 0.3 |
| Boiled E1 | Boiled E3 | 165.0 | 1 | 0.5 |
| Boiled E2 | Boiled E3 | 169.0 | 1 | 1.0 |

Bolded rows indicate significance at p ≤ 0.002 following a Bonferroni correction. This table corresponds to letters denoting significance in Figure 4B of the main text.

**Supplemental Table 7: Pairwise comparisons of percent conidia germination following a Generalized linear mixed effects model for the intra-chorionic experiment.**

| **Comparison** | | **T-value** | **df** | **p-value** |
| --- | --- | --- | --- | --- |
| **Unboiled E1** | **Unboiled E2** | **6.2** | **14288** | **< 0.001** |
| **Unboiled E1** | **Unboiled E3** | **10.9** | **14288** | **< 0.001** |
| **Unboiled E1** | **Boiled E1** | **4.9** | **14288** | **< 0.001** |
| Unboiled E1 | Boiled E2 | 1.3 | 14288 | 0.2 |
| **Unboiled E1** | **Boiled E3** | **4.7** | **14288** | **< 0.001** |
| **Unboiled E2** | **Unboiled E3** | **6.9** | **14288** | **< 0.001** |
| **Unboiled E2** | **Boiled E1** | **7.3** | **14288** | **< 0.001** |
| **Unboiled E2** | **Boiled E2** | **4.4** | **14288** | **< 0.001** |
| **Unboiled E2** | **Boiled E3** | **11.5** | **14288** | **< 0.001** |
| **Unboiled E3** | **Boiled E1** | **7.9** | **14288** | **< 0.001** |
| **Unboiled E3** | **Boiled E2** | **4.9** | **14288** | **< 0.001** |
| **Unboiled E3** | **Boiled E3** | **17.5** | **14288** | **< 0.001** |
| Boiled E1 | Boiled E2 | 2.5 | 14288 | 0.01 |
| Boiled E1 | Boiled E3 | -1.8 | 14288 | 0.1 |
| Boiled E2 | Boiled E3 | 1.4 | 14288 | 0.2 |

Bolded rows indicate significance at p ≤ 0.003 following a Bonferroni correction.

**
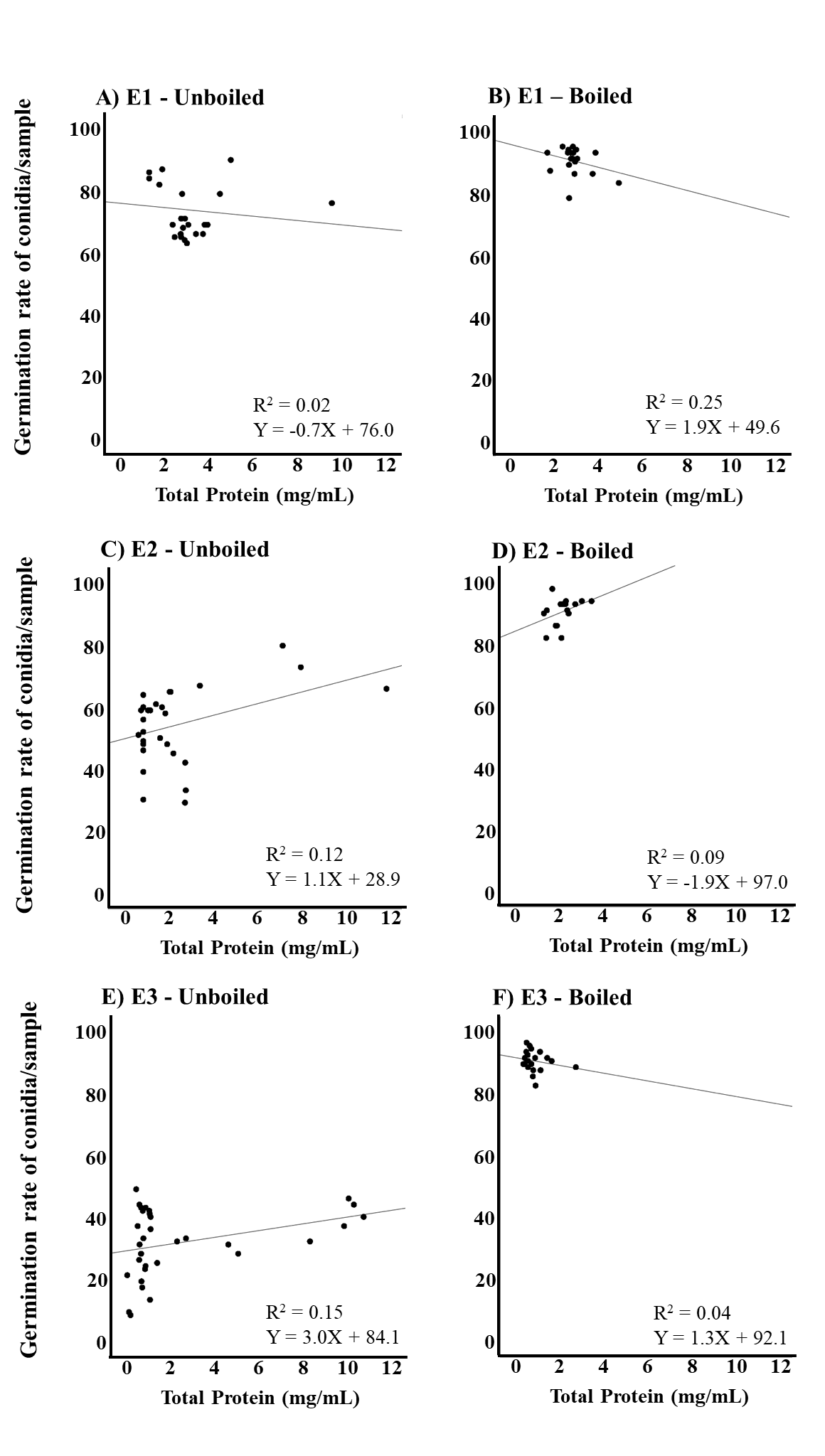
**

**Supplemental Figure 2: Germination rate of Conidia as a function of Total protein concentration across all treatments: A)** E1 unboiled, **B)** E1 boiled, **C)** E2 unboiled, **D)** E2 boiled, **E)** E3 unboiled, **F)** E3 boiled.

**Supplemental Material and Data 2: Protein Content of Extra-chorionic Washes & Intra-chorionic Contents.**

E1 extra-chorionic washes had ~ 2.5 mg/mL more protein than the corresponding extra-chorionic washes of E2 and E3 embryos (z = -4.2, df = 1, p < 0.001; z = -4.5, df = 1, p < 0.001 respectively; MW). Washes of the latter two stages had similar levels of protein (z = -0.4, df = 1, p = 0.7; MW). Extra-chorionic washes had significantly more soluble protein content than that of intra-chorionic samples (χ^2^ = 82.8, df = 1, p < 0.001; KW). E1 extra-chorionic washes had 7.8 mg/mL more protein than E1 intra-chorionic contents (z = -5.4, df = 1, p < 0.001). E2 and E3 had 5.4 mg/mL (z = -5.7, df = 1, p < 0.001), and 4.9 mg/mL (z = -4.4, df = 1, p < 0.001) more protein than E2 and E3 intra-chorionic contents, respectively.
