# Supplementary material for: Young but not defenceless: antifungal activity during embryonic development of a social insect
Source: R Soc Open Sci. 2020 Aug 26;7(8):191418. doi: 10.1098/rsos.191418 (PMC7481685; doi:10.1098/rsos.191418)
Supplement: Reviewer comments [file rsos191418_review_history.pdf]

THE ROYAL SOCIETY
PUBLISHING

ROYAL SOCIETY
OPEN SCIENCE

Young but not defenceless: antifungal activity during embryonic development of a social insect

Erin L. Cole, Haley Bayne and Rebeca B. Rosengaus

Article citation details
R. Soc. open sci. **7**: 191418.
http://dx.doi.org/10.1098/rsos.191418

Review timeline

Original submission:	16 January 2020
1st revised submission:	27 May 2020
2nd revised submission:	20 July 2020
Final acceptance:	27 July 2020

Note: Reports are unedited and appear as submitted by the referee. The review history appears in chronological order.

Note: This manuscript was transferred from another Royal Society journal with peer review.

Review History

RSOS-191418.R0 (Original submission)

Review form: Reviewer 1

Is the manuscript scientifically sound in its present form?
Yes

Are the interpretations and conclusions justified by the results?
Yes

Is the language acceptable?
Yes

Do you have any ethical concerns with this paper?
No

Have you any concerns about statistical analyses in this paper?
Yes

Recommendation?
Major revision is needed (please make suggestions in comments)

Reports © 2020 The Reviewers; Decision Letters © 2020 The Reviewers and Editors; Responses © 2020 The Reviewers, Editors and Authors. Published by the Royal Society under the terms of the Creative Commons Attribution License http://creativecommons.org/licenses/by/4.0/, which permits unrestricted use, provided the original author and source are credited

Comments to the Author(s)
I found the data very interesting an the approach original. I decided to read the manuscript before reading the comments of the previous reviewers in order not to be biased, and after checking saw that my comments did not seem to overlap with previous ones.
I enjoyed reading the manuscript, but sometimes found it lacked fluidity for reasons that can easily be addressed.

First, there is a detail that is unclear to me :
Since conidia of Metarhizium take less than 24 hours to germinate, and are able to germinate on the surface of termite eggs, one could wonder whether they have germinated during the incubation with egg/embryo extracts instead of after plating on PDA. Do the authors have any data or indication so as to when the main germination events take place ? Were the hyphae longer in some treatments compared to control ?

- I found the denomination "embryo" to refer to eggs confusing. Is there a good reason for not simply referring to them as eggs ?

- Starting from line 170, of the treatments applied to the eggs to assess the activity of different parts of the chorion, it took me a long time to sort out what treatment was supposed to expose what part of the egg, or what fraction the authors were isolating and why.
The first experiment basically assesses the fungistatic activity of the surface of the eggs, while the second assesses the activity of the inside, i.e. the embryo and the inner part of the chorion if I understood correctly.
- Line 217 : does the sonication result in the homogenization of the egg content into the solution ? Does it remove the outer layer of the chorion ? From the figure, I understand a cell lysis is happening, but of which tissue and to what extent ?

- Line 203 : even if the PI was inhibiting conidia germination, it is the proper control.

- Line 226 : the statistics section is a bit messy, due to the fact that the authors seem to have carried redundant analyses. This also makes the reading of the results difficult, and at times of the discussion. Such sentences as "even after correcting for COO" could be avoided if accounting for COO was always done. Considering all COO do not represent an equal number of embryonic stages, controlling for COO is obviously important.
And since GLMMs could be fitted to the data, and controlling for the colony of origin is the proper way to analyse it, the non parametric tests are useless. I would advise removing any mention of them. With what distribution were the GLMM fitted ? My guess would be Poisson but this needs to be stated somewhere in the stats section.
Protein content was normally distributed, and if the residuals also were after fitting a Linear Mixed Model, this could have been used instead of a GLMM. Maybe it is what the authors did and this is just a confusion in the statistics section.

- Line 240, rates are indeed not normally distributed.
More importantly, they are bound between 0 and 1, and therefore do not meet the criteria required for analyzing them with a linear model.
I would recommend using a a beta regression, with which the data has to be comprised between 0 and 1 without being 0 or 1, as there seems from Figure 5 that there are no 100 or 0 percent germination rates.
Alternatively, the authors could use a GLMM fitted for a binomial distribution, one column of the dataset stating the number of germinated conidia, the other column the number of non germinated conidia, and then using the equivalent of the cbind function in R with SPSS.

Supplemental Table 6 : I do not think you can use a t-test here.

Line 248 : The only way to know if total protein content does not affect fungistasis is to include it as an explanatory variable as an interaction with embryonic stage and whether or not the sample was boiled, and test whether this three way interaction is significant or not... I would expect for example that protein content has no effect on fungistasis in samples where you denaturated the proteins, but one can reasonably expect it to have an effect in sample where proteins are intact.
It is possible that the variance in protein content within one embryonic stage is not high enough to allow for such a model to run, in which case I would state it somewhere, and run one model with embryonic stage as a covariate (as the authors did) and one model with protein content as a covariate, and compare the results of both models.
Also, I think the supplemental figure 2 should be integrated in the main results, when the authors talk about the protein content relative to their staging of the embryos.

Results section :
Line 257, what do the authors mean by independent predictor ?

The use of colors in boxplots is nice, but it should be indicated what they correspond to in the legend.

In the discussion, I think there is an alternative hypothesis , please correct me if I am wrong :
While conidia germinate at the surface of an egg or cuticle, it is not the conidia which would naturally find itself in contact with the inside of an egg, but the penetration peg/mycelium/hyphal bodies. Is it possible that instead of a direct antifungal activity that would have been selected in the host, there is an inhibition of conidial germination that would have been selected in the fungus when the environment is not suitable ?

I am not remaining anonymous in case the authors would like more clarifications on my comments. I hope they help, and I would like to apologize again for the delay !

Review form: Reviewer 2

Is the manuscript scientifically sound in its present form?
Yes

Are the interpretations and conclusions justified by the results?
No

Is the language acceptable?
Yes

Do you have any ethical concerns with this paper?
No

Have you any concerns about statistical analyses in this paper?
No

Recommendation?
Major revision is needed (please make suggestions in comments)

Comments to the Author(s)
The manuscript tests the role of termite extraembryonic washes and intraembryonic contents against the fungus Metarhizium. The authors characterize stages of embryonic growth and demonstrate that extraembryonic washes have weak but a statistically significant effect on fungal

viability, whereas the effect is more pronounced with embryonic homogenates. Although the study is interesting, I have a few concerns, which are outlined as follows.

1. It is not obvious, which termite species is used in the manuscript until much later. Ideally, the name of the species can be stated in the abstract so that the results can be placed clearly in the context of the study system.
2. The authors report that extra-embryonic washes also reduced conidia viability, and based on their results, they interpret the active compound to be proteinaceous. Were the egg washes also analyzed using SDS-PAGE? A comparative analysis of the protein content of the washes and the intra-embryonic content could allow speculating on the possible sources of the chorionic antifungal proteins. Currently there is no way to know if these proteins are produced by the embryo itself or come from adult termite secretions or applications.
3. In addition, the current experimental setup does not allow for testing if the same proteins are involved in extraembryonic and intraembryonic antifungal defense.
4. The authors refer to vitellogenin as a potential (but unlikely) candidate for mediating intra-embryonic antifungal activity (e.g. lines 335-345). Although the molecular weight of vitellogenins can be variable, they are fairly large molecules. However, Figure 1 indicates that the most dominant bands in embryo extracts were < 100 kDa. What is the molecular weight of the subunits of vitellogenin in the species? Did the authors run a SDS-PAGE with egg homogenates as a positive control to identify a similar corresponding protein band? Were there any other assays to establish vitellogenin identity?
5. Overall, the results of the SDS-PAGE analysis of embryonic extracts are short and uninformative. For example, it is not clear how the authors infer that "protein profiles validated" and "accurately reflected physiologically distinct embryonic stages". These results need further elaboration, which criteria were used to infer differences? There is no validation or independent characterization of the identity or function of the constituent protein bands. Without these, there is very little inference that can be drawn from the single SDS PAGE.
6. The result that intraembryonic extracts have more potent antifungal activity than extraembryonic washes needs to be discussed in detail. Do the authors hypothesize that the embryo needing stronger intra-embryonic protection against fungi than external defenses? The authors state that Metarhizium binds and germinates on the outer surface of embryos. If this is true and if the fungus is originating from the soil or is present in the embryonic vicinity, how is the weak antifungal activity of embryonic washes explained. The implications of these results are not clear currently. Is there any information if Metarhizum invades the embryonic chorion or the serosa? If not, what is the justification for testing antifungal activity of intraembryonic contents? The implications and possible causes for a strong intraembryonic antifungal defense need to be discussed further.

Decision letter (RSOS-191418.R0)

16-Mar-2020

Dear Ms Cole,

The editors assigned to your paper ("Young but not defenseless: Antifungal activity during embryonic development of a social insect") have now received comments from reviewers. We would like you to revise your paper in accordance with the referee and Associate Editor suggestions which can be found below (not including confidential reports to the Editor). Please note this decision does not guarantee eventual acceptance.

Please submit a copy of your revised paper before 08-Apr-2020. Please note that the revision deadline will expire at 00.00am on this date. If we do not hear from you within this time then it will be assumed that the paper has been withdrawn. In exceptional circumstances, extensions may be possible if agreed with the Editorial Office in advance. We do not allow multiple rounds

of revision so we urge you to make every effort to fully address all of the comments at this stage. If deemed necessary by the Editors, your manuscript will be sent back to one or more of the original reviewers for assessment. If the original reviewers are not available, we may invite new reviewers.

To revise your manuscript, log into http://mc.manuscriptcentral.com/rsos and enter your Author Centre, where you will find your manuscript title listed under "Manuscripts with Decisions." Under "Actions," click on "Create a Revision." Your manuscript number has been appended to denote a revision. Revise your manuscript and upload a new version through your Author Centre.

When submitting your revised manuscript, you must respond to the comments made by the referees and upload a file "Response to Referees" in "Section 6 - File Upload". Please use this to document how you have responded to the comments, and the adjustments you have made. In order to expedite the processing of the revised manuscript, please be as specific as possible in your response.

In addition to addressing all of the reviewers' and editor's comments please also ensure that your revised manuscript contains the following sections as appropriate before the reference list:

• Ethics statement (if applicable)
If your study uses humans or animals please include details of the ethical approval received, including the name of the committee that granted approval. For human studies please also detail whether informed consent was obtained. For field studies on animals please include details of all permissions, licences and/or approvals granted to carry out the fieldwork.

• Data accessibility
It is a condition of publication that all supporting data are made available either as supplementary information or preferably in a suitable permanent repository. The data accessibility section should state where the article's supporting data can be accessed. This section should also include details, where possible of where to access other relevant research materials such as statistical tools, protocols, software etc can be accessed. If the data have been deposited in an external repository this section should list the database, accession number and link to the DOI for all data from the article that have been made publicly available. Data sets that have been deposited in an external repository and have a DOI should also be appropriately cited in the manuscript and included in the reference list.

If you wish to submit your supporting data or code to Dryad (http://datadryad.org/), or modify your current submission to dryad, please use the following link:
http://datadryad.org/submit?journalID=RSOS&manu=RSOS-191418

• Competing interests
Please declare any financial or non-financial competing interests, or state that you have no competing interests.

• Authors' contributions
All submissions, other than those with a single author, must include an Authors' Contributions section which individually lists the specific contribution of each author. The list of Authors should meet all of the following criteria; 1) substantial contributions to conception and design, or acquisition of data, or analysis and interpretation of data; 2) drafting the article or revising it critically for important intellectual content; and 3) final approval of the version to be published.

All contributors who do not meet all of these criteria should be included in the acknowledgements.

We suggest the following format:
AB carried out the molecular lab work, participated in data analysis, carried out sequence alignments, participated in the design of the study and drafted the manuscript; CD carried out the statistical analyses; EF collected field data; GH conceived of the study, designed the study, coordinated the study and helped draft the manuscript. All authors gave final approval for publication.

• Acknowledgements
Please acknowledge anyone who contributed to the study but did not meet the authorship criteria.

• Funding statement
Please list the source of funding for each author.

Once again, thank you for submitting your manuscript to Royal Society Open Science and I look forward to receiving your revision. If you have any questions at all, please do not hesitate to get in touch.

Kind regards,
Anita Kristiansen
Editorial Coordinator

Royal Society Open Science
openscience@royalsociety.org

on behalf of Kevin Padian (Subject Editor)
openscience@royalsociety.org

Associate Editor's comments:
Comments to the Author:
Thank you for submitting this manuscript to Royal Society Open Science. As you'll see, the reviewers consulted have a number of comments you'll need to address in any revision. Please take care to carefully respond to their queries in your revision, and a point-by-point response, too.

Comments to Author:

Reviewers' Comments to Author:
Reviewer: 1

Comments to the Author(s)
I found the data very interesting an the approach original. I decided to read the manuscript before reading the comments of the previous reviewers in order not to be biased, and after checking saw that my comments did not seem to overlap with previous ones.
I enjoyed reading the manuscript, but sometimes found it lacked fluidity for reasons that can easily be addressed.

First, there is a detail that is unclear to me :
Since conidia of Metarhizium take less than 24 hours to germinate, and are able to germinate on the surface of termite eggs, one could wonder whether they have germinated during the incubation with egg/embryo extracts instead of after plating on PDA. Do the authors have any data or indication so as to when the main germination events take place ? Were the hyphae longer in some treatments compared to control ?

- I found the denomination “embryo” to refer to eggs confusing. Is there a good reason for not simply referring to them as eggs ?

- Starting from line 170, of the treatments applied to the eggs to assess the activity of different parts of the chorion, it took me a long time to sort out what treatment was supposed to expose what part of the egg, or what fraction the authors were isolating and why.
The first experiment basically assesses the fungistatic activity of the surface of the eggs, while the second assesses the activity of the inside, i.e. the embryo and the inner part of the chorion if I understood correctly.
- Line 217 : does the sonication result in the homogenization of the egg content into the solution ? Does it remove the outer layer of the chorion ? From the figure, I understand a cell lysis is happening, but of which tissue and to what extent ?

- Line 203 : even if the PI was inhibiting conidia germination, it is the proper control.

- Line 226 : the statistics section is a bit messy, due to the fact that the authors seem to have carried redundant analyses. This also makes the reading of the results difficult, and at times of the discussion. Such sentences as "even after correcting for COO" could be avoided if accounting for COO was always done. Considering all COO do not represent an equal number of embryonic stages, controlling for COO is obviously important.
And since GLMMs could be fitted to the data, and controlling for the colony of origin is the proper way to analyse it, the non parametric tests are useless. I would advise removing any mention of them. With what distribution were the GLMM fitted ? My guess would be Poisson but this needs to be stated somewhere in the stats section.
Protein content was normally distributed, and if the residuals also were after fitting a Linear Mixed Model, this could have been used instead of a GLMM. Maybe it is what the authors did and this is just a confusion in the statistics section.

- Line 240, rates are indeed not normally distributed.
More importantly, they are bound between 0 and 1, and therefore do not meet the criteria required for analyzing them with a linear model.
I would recommend using a a beta regression, with which the data has to be comprised between 0 and 1 without being 0 or 1, as there seems from Figure 5 that there are no 100 or 0 percent germination rates.
Alternatively, the authors could use a GLMM fitted for a binomial distribution, one column of the dataset stating the number of germinated conidia, the other column the number of non germinated conidia, and then using the equivalent of the cbind function in R with SPSS.

Supplemental Table 6 : I do not think you can use a t-test here.

Line 248 : The only way to know if total protein content does not affect fungistasis is to include it as an explanatory variable as an interaction with embryonic stage and whether or not the sample was boiled, and test whether this three way interaction is significant or not... I would expect for example that protein content has no effect on fungistasis in samples where you denaturated the proteins, but one can reasonably expect it to have an effect in sample where proteins are intact.
It is possible that the variance in protein content within one embryonic stage is not high enough to allow for such a model to run, in which case I would state it somewhere, and run one model with embryonic stage as a covariate (as the authors did) and one model with protein content as a covariate, and compare the results of both models.
Also, I think the supplemental figure 2 should be integrated in the main results, when the authors talk about the protein content relative to their staging of the embryos.

Results section :
Line 257, what do the authors mean by independent predictor ?

The use of colors in boxplots is nice, but it should be indicated what they correspond to in the legend.

In the discussion, I think there is an alternative hypothesis , please correct me if I am wrong : While conidia germinate at the surface of an egg or cuticle, it is not the conidia which would naturally find itself in contact with the inside of an egg, but the penetration peg/mycelium/hyphal bodies. Is it possible that instead of a direct antifungal activity that would have been selected in the host, there is an inhibition of conidial germination that would have been selected in the fungus when the environment is not suitable ?

I am not remaining anonymous in case the authors would like more clarifications on my comments. I hope they help, and I would like to apologize again for the delay !

Reviewer: 2

Comments to the Author(s)
The manuscript tests the role of termite extraembryonic washes and intraembryonic contents against the fungus Metarhizium. The authors characterize stages of embryonic growth and demonstrate that extraembryonic washes have weak but a statistically significant effect on fungal viability, whereas the effect is more pronounced with embryonic homogenates. Although the study is interesting, I have a few concerns, which are outlined as follows.

1. It is not obvious, which termite species is used in the manuscript until much later. Ideally, the name of the species can be stated in the abstract so that the results can be placed clearly in the context of the study system.
2. The authors report that extra-embryonic washes also reduced conidia viability, and based on their results, they interpret the active compound to be proteinaceous. Were the egg washes also analyzed using SDS-PAGE? A comparative analysis of the protein content of the washes and the intra-embryonic content could allow speculating on the possible sources of the chorionic antifungal proteins. Currently there is no way to know if these proteins are produced by the embryo itself or come from adult termite secretions or applications.
3. In addition, the current experimental setup does not allow for testing if the same proteins are involved in extraembryonic and intraembryonic antifungal defense.
4. The authors refer to vitellogenin as a potential (but unlikely) candidate for mediating intra-embryonic antifungal activity (e.g. lines 335-345). Although the molecular weight of vitellogenins can be variable, they are fairly large molecules. However, Figure 1 indicates that the most dominant bands in embryo extracts were < 100 kDa. What is the molecular weight of the subunits of vitellogenin in the species? Did the authors run a SDS-PAGE with egg homogenates as a positive control to identify a similar corresponding protein band? Were there any other assays to establish vitellogenin identity?
5. Overall, the results of the SDS-PAGE analysis of embryonic extracts are short and uninformative. For example, it is not clear how the authors infer that “protein profiles validated” and “accurately reflected physiologically distinct embryonic stages”. These results need further elaboration, which criteria were used to infer differences? There is no validation or independent characterization of the identity or function of the constituent protein bands. Without these, there is very little inference that can be drawn from the single SDS PAGE.
6. The result that intraembryonic extracts have more potent antifungal activity than extraembryonic washes needs to be discussed in detail. Do the authors hypothesize that the embryo needing stronger intra-embryonic protection against fungi than external defenses? The authors state that Metarhizium binds and germinates on the outer surface of embryos. If this is true and if the fungus is originating from the soil or is present in the embryonic vicinity, how is the weak antifungal activity of embryonic washes explained. The implications of these results are not clear currently. Is there any information if Metarhizum invades the embryonic chorion or the serosa? If not, what is the justification for testing antifungal activity of intraembryonic contents? The implications and possible causes for a strong intraembryonic antifungal defense need to be discussed further.

Author's Response to Decision Letter for (RSOS-191418.R0)

See Appendix A.

RSOS-191418.R1 (Revision)

Review form: Reviewer 1

Is the manuscript scientifically sound in its present form?
Yes

Are the interpretations and conclusions justified by the results?
Yes

Is the language acceptable?
Yes

Do you have any ethical concerns with this paper?
No

Have you any concerns about statistical analyses in this paper?
Yes

Recommendation?
Accept with minor revision (please list in comments)

Comments to the Author(s)
First of all I would like to apologize for the delay I have caused in the reviewing process, but I had only sporadic internet access since I was moving in an other city.
I thank the authors for clarifying the aspects of the manuscripts that were difficult for me to understand. I only have a few additional comments left.

In the statistics section : it would be useful if the authors were stating exactly which model was run for the Kruskal Wallis test, similarly to what they did for the GLMM. If I understood correctly this was germination rate as a response variable as a function of presence of intact or boiled embryonic extract (three levels in the treatment variable : control, unboiled and boiled right ?).

For the GLMM, the treatment is actually a concatenation of two variables : the embryonic stage and whether the samples were boiled or not. But since the KW test revealed no differences in the germination rates of conidia incubated with boiled samples, these boiled samples can be seen as a control treatment. So then why not test for the interaction between embryonic stage and boiling/not boiling of the samples instead ?
This would make the reading of the results easier, since I suppose for example in Experiment 1 that E1 extra chorionic extracts have a fungistatic activity on conidia compared to E2, E3, and boiled extracts. E2 and E3 on the other hand should not reduce conidia viability compared to boiled extracts.

Line 283 is confusing : GLMM yielded similar results : the GLMM did not compare the embryonic washes/contents to control, and therefore cannot yield the same conclusion. My comment above might help clarify this.
I think instead of lines 283 to 289, the correct formulation would be "Embryonic stage has a significant effect on the viability of conidia incubated with extra-chorionic washes. Conidia incubated with E1 stage extra chorionic washes have a lower germination rate compared to conidia incubated with E2 and E3 stages (F = 2.1, df = 11, 13488, p =0.06, GLMM)."

Lines 302 to 305 : the discrepancy between the results of the model and the results of the post hoc does not indicate anything about the amount of variance explained by the variable. I would instead describe the interaction, and then say that these differences do not hold after Bonferroni correction.
Corrections for multiple comparisons are useful when analyzes do indeed require a lot of pairwise comparisons which increases the rate of false discovery, however they have often been criticized for randomly increasing p-values without regard for effect size. This might be the reason why the interaction does not show during the post hoc.

Review form: Reviewer 2

Is the manuscript scientifically sound in its present form?
Yes

Are the interpretations and conclusions justified by the results?
Yes

Is the language acceptable?
Yes

Do you have any ethical concerns with this paper?
No

Have you any concerns about statistical analyses in this paper?
No

Recommendation?
Accept as is

Comments to the Author(s)
Thank you for the detailed responses to all my comments.

Decision letter (RSOS-191418.R1)

We hope you are keeping well at this difficult and unusual time. We continue to value your support of the journal in these challenging circumstances. If Royal Society Open Science can assist you at all, please don't hesitate to let us know at the email address below.

Dear Ms Cole:

On behalf of the Editors, I am pleased to inform you that your Manuscript RSOS-191418.R1 entitled "Young but not defenseless: Antifungal activity during embryonic development of a

social insect" has been accepted for publication in Royal Society Open Science subject to minor revision in accordance with the referee suggestions. Please find the referees' comments at the end of this email.

The reviewers and Subject Editor have recommended publication, but also suggest some minor revisions to your manuscript. Therefore, I invite you to respond to the comments and revise your manuscript.

• Ethics statement
If your study uses humans or animals please include details of the ethical approval received, including the name of the committee that granted approval. For human studies please also detail whether informed consent was obtained. For field studies on animals please include details of all permissions, licences and/or approvals granted to carry out the fieldwork.

• Data accessibility
It is a condition of publication that all supporting data are made available either as supplementary information or preferably in a suitable permanent repository. The data accessibility section should state where the article's supporting data can be accessed. This section should also include details, where possible of where to access other relevant research materials such as statistical tools, protocols, software etc can be accessed. If the data has been deposited in an external repository this section should list the database, accession number and link to the DOI for all data from the article that has been made publicly available. Data sets that have been deposited in an external repository and have a DOI should also be appropriately cited in the manuscript and included in the reference list.

If you wish to submit your supporting data or code to Dryad (http://datadryad.org/), or modify your current submission to dryad, please use the following link:
http://datadryad.org/submit?journalID=RSOS&manu=RSOS-191418.R1

• Competing interests
Please declare any financial or non-financial competing interests, or state that you have no competing interests.

• Authors' contributions
All submissions, other than those with a single author, must include an Authors' Contributions section which individually lists the specific contribution of each author. The list of Authors should meet all of the following criteria; 1) substantial contributions to conception and design, or acquisition of data, or analysis and interpretation of data; 2) drafting the article or revising it critically for important intellectual content; and 3) final approval of the version to be published.

All contributors who do not meet all of these criteria should be included in the acknowledgements.

We suggest the following format:
AB carried out the molecular lab work, participated in data analysis, carried out sequence alignments, participated in the design of the study and drafted the manuscript; CD carried out the statistical analyses; EF collected field data; GH conceived of the study, designed the study, coordinated the study and helped draft the manuscript. All authors gave final approval for publication.

• Acknowledgements
Please acknowledge anyone who contributed to the study but did not meet the authorship criteria.

• Funding statement
Please list the source of funding for each author.

Please note that we cannot publish your manuscript without these end statements included. We have included a screenshot example of the end statements for reference. If you feel that a given heading is not relevant to your paper, please nevertheless include the heading and explicitly state that it is not relevant to your work.

Because the schedule for publication is very tight, it is a condition of publication that you submit the revised version of your manuscript before 22-Jul-2020. Please note that the revision deadline will expire at 00.00am on this date. If you do not think you will be able to meet this date please let me know immediately.

To revise your manuscript, log into https://mc.manuscriptcentral.com/rsos and enter your Author Centre, where you will find your manuscript title listed under "Manuscripts with Decisions". Under "Actions," click on "Create a Revision." You will be unable to make your revisions on the originally submitted version of the manuscript. Instead, revise your manuscript and upload a new version through your Author Centre.

When submitting your revised manuscript, you will be able to respond to the comments made by the referees and upload a file "Response to Referees" in "Section 6 - File Upload". You can use this to document any changes you make to the original manuscript. In order to expedite the processing of the revised manuscript, please be as specific as possible in your response to the referees.

When uploading your revised files please make sure that you have:

1) A text file of the manuscript (tex, txt, rtf, docx or doc), references, tables (including captions) and figure captions. Do not upload a PDF as your "Main Document".
2) A separate electronic file of each figure (EPS or print-quality PDF preferred (either format should be produced directly from original creation package), or original software format)
3) Included a 100 word media summary of your paper when requested at submission. Please ensure you have entered correct contact details (email, institution and telephone) in your user account
4) Included the raw data to support the claims made in your paper. You can either include your data as electronic supplementary material or upload to a repository and include the relevant doi within your manuscript
5) All supplementary materials accompanying an accepted article will be treated as in their final form. Note that the Royal Society will neither edit nor typeset supplementary material and it will be hosted as provided. Please ensure that the supplementary material includes the paper details where possible (authors, article title, journal name).

Supplementary files will be published alongside the paper on the journal website and posted on the online figshare repository (https://figshare.com). The heading and legend provided for each supplementary file during the submission process will be used to create the figshare page, so please ensure these are accurate and informative so that your files can be found in searches. Files on figshare will be made available approximately one week before the accompanying article so that the supplementary material can be attributed a unique DOI.

Once again, thank you for submitting your manuscript to Royal Society Open Science and I look forward to receiving your revision. If you have any questions at all, please do not hesitate to get in touch.

Kind regards,
Royal Society Open Science Editorial Office
Royal Society Open Science
openscience@royalsociety.org

on behalf of Prof Kevin Padian (Subject Editor)
openscience@royalsociety.org

Associate Editor Comments to Author:
The reviewers have a number of minor changes for you to tackle, but these should be relatively straightforward to implement.

Reviewer comments to Author:
Reviewer: 2

Comments to the Author(s)
Thank you for the detailed responses to all my comments.

Reviewer: 1

Comments to the Author(s)
First of all I would like to apologize for the delay I have caused in the reviewing process, but I had only sporadic internet access since I was moving in an other city.
I thank the authors for clarifying the aspects of the manuscripts that were difficult for me to understand. I only have a few additional comments left.

In the statistics section : it would be useful if the authors were stating exactly which model was run for the Kruskal Wallis test, similarly to what they did for the GLMM. If I understood correctly this was germination rate as a response variable as a function of presence of intact or boiled embryonic extract (three levels in the treatment variable : control, unboiled and boiled right ?).

For the GLMM, the treatment is actually a concatenation of two variables : the embryonic stage and whether the samples were boiled or not. But since the KW test revealed no differences in the germination rates of conidia incubated with boiled samples, these boiled samples can be seen as a control treatment. So then why not test for the interaction between embryonic stage and boiling/not boiling of the samples instead ?
This would make the reading of the results easier, since I suppose for example in Experiment 1 that E1 extra chorionic extracts have a fungistatic activity on conidia compared to E2, E3, and boiled extracts. E2 and E3 on the other hand should not reduce conidia viability compared to boiled extracts.

Line 283 is confusing : GLMM yielded similar results : the GLMM did not compare the embryonic washes/contents to control, and therefore cannot yield the same conclusion. My comment above might help clarify this.
I think instead of lines 283 to 289, the correct formulation would be "Embryonic stage has a significant effect on the viability of conidia incubated with extra-chorionic washes. Conidia incubated with E1 stage extra chorionic washes have a lower germination rate compared to conidia incubated with E2 and E3 stages ($F = 2.1$, $df = 11, 13488$, $p = 0.06$, GLMM)."

Lines 302 to 305 : the discrepancy between the results of the model and the results of the post hoc does not indicate anything about the amount of variance explained by the variable. I would instead describe the interaction, and then say that these differences do not hold after Bonferroni correction.
Corrections for multiple comparisons are useful when analyzes do indeed require a lot of pairwise comparisons which increases the rate of false discovery, however they have often been criticized for randomly increasing p-values without regard for effect size. This might be the reason why the interaction does not show during the post hoc.

Author's Response to Decision Letter for (RSOS-191418.R1)

See Appendix B.

Decision letter (RSOS-191418.R2)

We hope you are keeping well at this difficult and unusual time. We continue to value your support of the journal in these challenging circumstances. If Royal Society Open Science can assist you at all, please don't hesitate to let us know at the email address below.

Dear Dr Cole,

It is a pleasure to accept your manuscript entitled "Young but not defenseless: Antifungal activity during embryonic development of a social insect" in its current form for publication in Royal Society Open Science.

You can expect to receive a proof of your article in the near future. Please contact the editorial office (openscience_proofs@royalsociety.org) and the production office (openscience@royalsociety.org) to let us know if you are likely to be away from e-mail contact -- if you are going to be away, please nominate a co-author (if available) to manage the proofing process, and ensure they are copied into your email to the journal.

Due to rapid publication and an extremely tight schedule, if comments are not received, your paper may experience a delay in publication. Royal Society Open Science operates under a continuous publication model. Your article will be published straight into the next open issue and this will be the final version of the paper. As such, it can be cited immediately by other researchers. As the issue version of your paper will be the only version to be published I would advise you to check your proofs thoroughly as changes cannot be made once the paper is published.

Please see the Royal Society Publishing guidance on how you may share your accepted author manuscript at https://royalsociety.org/journals/ethics-policies/media-embargo/.

Thank you for your fine contribution. On behalf of the Editors of Royal Society Open Science, we look forward to your continued contributions to the Journal.

Kind regards,
Andrew Dunn
Royal Society Open Science Editorial Office
Royal Society Open Science
openscience@royalsociety.org

on behalf of Prof Kevin Padian (Subject Editor)
openscience@royalsociety.org

Follow Royal Society Publishing on Twitter: @RSocPublishing
Follow Royal Society Publishing on Facebook:
https://www.facebook.com/RoyalSocietyPublishing.FanPage/
Read Royal Society Publishing's blog: https://blogs.royalsociety.org/publishing/

Appendix A

Mycosis Reviewer Feedback

Author Responses are given in Purple

General

• Ethics statement (if applicable) Not applicable as study uses insects

• Data accessibility 3 Excel files containing the data have been uploaded as supporting supplementary material

If you wish to submit your supporting data or code to Dryad (http://datadryad.org/), or modify your current submission to dryad, please use the following link:

http://datadryad.org/submit?journalID=RSOS&manu=RSOS-191418

I have submitted the data files to https://doi.org/10.5061/dryad.h18931zh3. However, given our previous issues with Dryad, these files will remain as supplemental files uploaded along with the manuscript until I have confirmation from both Dryad and the editors of RSOS that this doi link works.

• Competing interests We have added a statement declaring no competing interests- Line 367

• Authors' contributions. All submissions, other than those with a single author, must include an Authors' Contributions section which individually lists the specific contribution of each author. The list of Authors should meet all of the following criteria; 1) substantial contributions to conception and design, or acquisition of data, or analysis and interpretation of data; 2) drafting the article or revising it critically for important intellectual content; and 3) final approval of the version to be published.

All contributors who do not meet all of these criteria should be included in the acknowledgements.

We have discussed authorship with all original authors. All, including Jeremy McDavid, have agreed with this new list of authors. Given your journal's guidelines, Jeremy McDavid's name was removed as he only contributed to the early stages of the research. We have included his name in the revised acknowledgements section. We do have an email form Mr. McDavid stating he is ok with our authorship changes, in case the journal wants to see it.

• Acknowledgements already included, line 393

• Funding statement Modified acknowledgments to have a separate funding statement, now line 403.

Associate Editor's comments:

Comments to the Author:

Thank you for submitting this manuscript to Royal Society Open Science. As you'll see, the reviewers consulted have a number of comments you'll need to address in any revision. Please take care to carefully respond to their queries in your revision, and a point-by-point response, too.

We thank the Associate Editor and Reviewers for their time and feedback. We have revised our manuscript according to their recommendations, and all revisions are highlighted in yellow in the new version. We provide point-by-point responses to both Reviewers below.

Reviewer: 1

Comments to the Author(s):

I found the data very interesting and the approach original. I decided to read the manuscript before reading the comments of the previous reviewers in order not to be biased, and after checking saw that my comments did not seem to overlap with previous ones.

I enjoyed reading the manuscript, but sometimes found it lacked fluidity for reasons that can easily be addressed.

We thank Reviewer 1 for his/her time and feedback. We have addressed each of your concerns below and revised the manuscript accordingly. All edits on the ms have been highlighted in yellow.

First, there is a detail that is unclear to me:

Since conidia of Metarhizium take less than 24 hours to germinate and are able to germinate on the surface of termite eggs, one could wonder whether they have germinated during the incubation with egg/embryo extracts instead of after plating on PDA. Do the authors have any data or indication so as to when the main germination events take place? Were the hyphae longer in some treatments compared to control?

We observed no germination during the incubation with the embryo washes or homogenates. All germination took place only after conidia was seeded on the PDA medium, which provides the optimal conditions for conidia germination (see citations 28, 49 in the main text). We have now added two sentences about this on lines 184 and 189 of the revision. Hyphae were not measured as a part of this study but could represent a novel and interesting area for future research.

- I found the denomination “embryo” to refer to eggs confusing. Is there a good reason for not simply referring to them as eggs?

The term “embryo” was suggested by a colleague Dr. Phyllis Strauss of Northeastern University who read an earlier version of our manuscript. Her reasoning was that eggs are unfertilized gametes, while embryos describe presumed fertilized eggs. Throughout our ms, we assume that oviposited eggs were indeed diploid fertilized entities, and thus, they actually represent

developing embryos rather than unfertilized female gametes (“eggs”). Although we see your point, we have opted to continue referring to the fertilized eggs as embryos. We have explain our rationale for the use of embryos (instead of eggs) on line 91-94 which now read: “We, henceforth, refer to the termite eggs as “embryos” to draw a distinction between the haploid female gamete (“egg”) and the diploid (fertilized and oviposited) developing eggs (“embryos”). We assumed all oviposited eggs were fertilized.”

- Starting from line 170, of the treatments applied to the eggs to assess the activity of different parts of the chorion, it took me a long time to sort out what treatment was supposed to expose what part of the egg, or what fraction the authors were isolating and why.

The first experiment basically assesses the fungistatic activity of the surface of the eggs, while the second assesses the activity of the inside, i.e. the embryo and the inner part of the chorion if I understood correctly.

The referee has understood correctly whether we were testing the fungistasis on the outer surface of the chorion (formerly extraembryonic) or within the chorion (formerly intraembryonic). To reduce any misunderstandings, we have changed these terms to “extra-chorionic” and “intra-chorionic” throughout the ms. These terms better reflect our experiments, given that the chorion itself is the natural barrier between the surrounding environment and the developing embryo. We hope these changes make it easier to follow our experimental design.

- Line 217 : does the sonication result in the homogenization of the egg content into the solution ? Does it remove the outer layer of the chorion? From the figure, I understand a cell lysis is happening, but of which tissue and to what extent?

Sonication ruptures the entire egg (including the chorion), which results in the spillage of the intra-chorionic contents into solution. We visually confirmed that the chorion was ruptured as the solution became opaque (i.e., “milky”) after sonication. Following sonication, the sample was centrifuged gently to pull down any larger remnants of the chorion. Hence the turbid supernatant presumably contained components of the lysed cells within the chorion, while the broken up larger pieces of chorion sank to the bottom of microcentrifuge tube. The supernatant was then transferred to a fresh microcentrifuge tube, thus minimizing any remaining antifungal activity originating from the outer surface of the chorion. We remind the referee that for our intra-chorionic experiments, the outer surface of the chorion was first UVed, then washed and only then sonicated, so we are confident that our intra-chorionic protocols minimized any antifungal contributions originating from the chorion itself. We have added more detail to our Methods, lines 132-136, to ensure that the reader better visualizes our protocols. The section now reads: “For each sample, we visually confirmed that the chorion had ruptured and that the solution became opaque (i.e., “milky”) after sonication. Following sonication, the sample was then centrifuged gently to pull down any larger remnants of the chorion. The supernatant was then transferred to a fresh microcentrifuge tube, and immediately flash frozen in liquid nitrogen, then stored at -80 °C.”

- Line 203 : even if the PI was inhibiting conidia germination, it is the proper control.

We agree, although it was worth stating that our experimental control had no effect on conidia germination.

- Line 226 : the statistics section is a bit messy, due to the fact that the authors seem to have carried redundant analyses. This also makes the reading of the results difficult, and at times of the discussion. Such sentences as “even after correcting for COO” could be avoided if accounting for COO was always done. Considering all COO do not represent an equal number of embryonic stages, controlling for COO is obviously important.

We have made significant revisions to the statistical analysis section based on your feedback below. We have endeavored to make the methods as clear as possible in the revised manuscript.

And since GLMMs could be fitted to the data, and controlling for the colony of origin is the proper way to analyse it, the non-parametric tests are useless. I would advise removing any mention of them. With what distribution were the GLMM fitted ? My guess would be Poisson but this needs to be stated somewhere in the stats section. Protein content was normally distributed, and if the residuals also were after fitting a Linear Mixed Model, this could have been used instead of a GLMM. Maybe it is what the authors did and this is just a confusion in the statistics section.

We want to assure the referee that we never used **generalized** linear mixed models, but rather **general** linear mixed models. The acronym “GLMM” has been used for both classes of models. Since our revised ms reports results of both general and generalized models, we now reserve GLMM for the generalized mixed models and are now using the acronym LMM to represent “general linear mixed models.” These acronyms have been clearly defined in our statistical analyses section.

Because the residuals from the linear mixed effects model were normally distributed and LMMs are considered to be robust analyses, we have now removed the KW test of embryo volume as a function of developmental stage from the manuscript, as suggested by the referee. We also made minor changes to the text to make it easier to read. The LMM is fitted to a normal distribution, now stated in line 223 and 226.

- Line 240, rates are indeed not normally distributed.

More importantly, they are bound between 0 and 1, and therefore do not meet the criteria required for analyzing them with a linear model.

I would recommend using a beta regression, with which the data has to be comprised between 0 and 1 without being 0 or 1, as there seems from Figure 5 that there are no 100 or 0 percent germination rates.

Alternatively, the authors could use a GLMM fitted for a binomial distribution, one column of the dataset stating the number of germinated conidia, the other column the number of non-germinated conidia, and then using the equivalent of the cbind function in R with SPSS.

We appreciate the detailed recommendation from Reviewer 1. Indeed, we agree, and have made significant revisions to the analysis, and manuscript. To make these changes easy to follow, we have provided a step by step list of each change, along with a rationale.

a. First, we ran a Kruskal-Wallis tests, along with post-hoc comparisons of all treatments (E1-unboiled, E2-unboiled, E3-unboiled, E1-boiled, E2-boiled, E3-boiled), **to test the hypothesis that conidia incubated with embryonic contents had lower germination rates than conidia grown without embryonic contents (controls).** A separate test was run for each of the extra- and intra-chorionic experiments. Although KW tests cannot control for COO, this step was necessary as the control conidia inherently lack any information regarding embryonic stage and colony of origin (COO). Thus, a GLMM cannot be used to test our hypothesis that incubation of conidia with extra-chorionic washes or intra-chorionic homogenates would reduce conidia germination relative to control conidia.

b. We used a GLMM to test **our second hypothesis: that conidia germination rates would differ as a function of the embryonic stage.** To reliably test this, we needed to control for COO, the total protein content of the extra-chorionic washes and that of the intra-chorionic homogenates. As before, a separate GLMM was run for the extra- and intra-chorionic experiments.

c. Following the reviewer's recommendations, we ran a Generalized linear mixed effects model using a binomial distribution and binary logit function. Treatment was entered as a fixed effect (E1-unboiled, E2-unboiled, E3-unboiled, E1-boiled, E2-boiled, E3-boiled). COO and Sample ID (see explanation below) were entered as random effects and total protein was entered as a covariate.

d. Although embryonic volume was also measured, this variable was positively correlated with protein content. Hence, we opted to include in the model only protein amount as this represents a more biologically meaningful variable.

e. SPSS lacks a cbind function equivalent. Thus, to perform this analysis, each germination rate (coded as a single data point for the KW test) had to be converted into a series of 0s and 1s representing each individual conidia: 0 = ungerminated, 1 = germinated. Hence, to account for the repeated measures, we listed Sample ID (of the original germination rate) as a random effect following the advice of a statistician in our department.

f. **CAVEAT:** Unfortunately, when the data was originally collected, it was collected directly as percentage number rather than the actual absolute conidia counts. Thus, we "recreated" these counts by converting each percent germination into 100 data points of the corresponding dummy variable (ungerminated = 0 and germinated = 1). This required rounding each percentage to the nearest integer. We believe this is a reasonable approximation of what the original conidia counts would have been while still allowing us to comply with the referee's statistical recommendation. Happily, the newest GLMM with a binomial distribution and a binary logit function and the previous analyses presented in our last submission are still very much in agreement. Using both tests, the GLMM (binomial distribution and binary logit function) and the KW, we feel our results and interpretation are robust despite the limitations of each individual analysis.

Supplemental Table 6 : I do not think you can use a t-test here.

Indeed, while the post-hoc tests use a t-statistic, they are not t-tests. These tables have been redone to reflect the new GLMMs (with binomial distribution and binary logit function) described above.

Results section:

Line 257, what do the authors mean by independent predictor ?

This line refers to the fact that embryonic stage (as a main effect) was significant after controlling for possible interactions and other factors (i.e., COO and total protein).

The use of colors in boxplots is nice, but it should be indicated what they correspond to in the legend.

We have added a legend.

In the discussion, I think there is an alternative hypothesis, please correct me if I am wrong :

While conidia germinate at the surface of an egg or cuticle, it is not the conidia which would naturally find itself in contact with the inside of an egg, but the penetration peg/mycelium/hyphal bodies. Is it possible that instead of a direct antifungal activity that would have been selected in the host, there is an inhibition of conidial germination that would have been selected in the fungus when the environment is not suitable?

Indeed, this is why we tested for both extra- and intra-chorionic antifungal activity. Also, as stated in line 364-366 of the discussion, the observed increase in antifungal activity of the intra-chorionic homogenates likely represents the development of the embryonic immune system – possibly in preparation for hatching and becoming mobile. Additional studies would need to be run in order to test whether intra-chorionic homogenates also negatively affect the development of more advanced stages of the fungus growth (e.g., hyphae and/or hyphael bodies). For those experiments, using blastospores, rather than conidia itself, would be advisable. Those experiments, although interesting, are beyond the scope of this study.

I am not remaining anonymous in case the authors would like more clarifications on my comments. I hope they help, and I would like to apologize again for the delay !

Thank you! Your reviewer comments regarding our statistics were very detailed and helpful! We know the quality and clarity of our ms was greatly improved by your constructive criticisms.

Reviewer: 2

Comments to the Author(s)

The manuscript tests the role of termite extraembryonic washes and intraembryonic contents against the fungus Metarhizium. The authors characterize stages of embryonic growth and demonstrate that extraembryonic washes have weak but a statistically significant effect on fungal

viability, whereas the effect is more pronounced with embryonic homogenates. Although the study is interesting, I have a few concerns, which are outlined as follows.

We thank Reviewer 2 for his/her time and feedback. We have addressed each of your concerns below and revised the manuscript accordingly. All edits on the ms have been highlighted in yellow.

1. It is not obvious, which termite species is used in the manuscript until much later. Ideally, the name of the species can be stated in the abstract so that the results can be placed clearly in the context of the study system.

We have corrected this oversight and included the full scientific name in line 22 (Abstract) and line 69 (Intro) of the revised ms.

2. The authors report that extra-embryonic washes also reduced conidia viability, and based on their results, they interpret the active compound to be proteinaceous. Were the egg washes also analyzed using SDS-PAGE? A comparative analysis of the protein content of the washes and the intra-embryonic content could allow speculating on the possible sources of the chorionic antifungal proteins. Currently there is no way to know if these proteins are produced by the embryo itself or come from adult termite secretions or applications.

The SDS-PAGE only analyzed homogenates of whole eggs. For this reason, the referee is correct in stating that "there is no way to know if these proteins are produced by the embryo itself or come from adult termite secretions or applications." There is a high probability that the protein profiles included compounds residing on the outer surface of the chorion such as proteins from the parents' salivary gland secretions. Although we agree that protein profiles of outside and inside of the egg would have been be ideal, such analysis is now beyond the scope of the current manuscript. We had previously addressed this limitation in lines 330 -333 of the original manuscript. Given the feedback of both current reviewers, we have removed the SDS-PAGE figure from our results section, and we revised the ms accordingly. It is important to point out that there are multiple studies demonstrating that termites lick their eggs and that their saliva has antifungal compounds (Rosengaus and Traniello, 1991; Cruse, 1998; Rosengaus et al, 2004; Bulmer et al., 2009; Hamilton, Lay and Bulmer, 2011; Rosengaus et al., 2014). Hence, the hypotheses as outlined in the discussion (lines 325-338; below) are reasonable:

"Extra-chorionic fungistasis, for example, could have resulted from either microbial byproducts, the deposition of salivary gland secretions during grooming by nestmates, and/or maternal coating/smearing during oviposition. Embryos receive intense brood care from kings, queens and workers (70 – 72). Such behaviors likely result in the physical removal of microbes from the surface of the chorion, a strategy akin to that reported during mutual grooming among workers (38). Embryo grooming/licking may also result in the deposition of salivary gland secretions on the chorion's exterior, which are known to possess antimicrobial properties (e.g., 44 – 45, 72). Specifically, active β-1,3-glucanases are excreted by the termite's salivary glands and are known to break down fungal cell walls (44). Further support for the role of salivary gland secretions containing antifungal compounds comes from the fact that E1 extra-chorionic washes had 2.5 mg/mL more protein than extra-chorionic washes of E2 and E3 (Supplemental Data 2).

Hence, the possibility exists that E1 are preferentially groomed/licked by parents/workers relative to E2 and E3 stages, and/or that the efficacy of the salivary antimicrobial compound(s) is temporary, waning as E1 embryos progress through their development."

3. In addition, the current experimental setup does not allow for testing if the same proteins are involved in extraembryonic and intraembryonic antifungal defense.

We agree this is a limitation of the current study. It is important to point out that our lab is not a proteomic lab. Our original aim was not to systematically identify and quantify the complete complement of proteins of termite embryos. Our aim was more modest: use an eco-immunology approach to establish if termite eggs exhibit antifungal properties given that entomopathogenic fungi pose significant selection pressures on other developmental stages of colonies. As stated in our previous responses to *Proceedings of the Royal Society B: Biological Sciences*, and now too to the Royal Society Open Science:

"We do agree that additional protein analysis (i.e., identification) would make for a more "complete story". However, we strongly believe that the data reported in the current manuscript is scientifically sound and of sufficient novelty to the growing fields of Ecological Immunology, in general, and Ecological Immunology of Social Insects in particular, to warrant publication in *Proceedings of the Royal Society B: Biological Sciences*. The lack of protein identification does not discredit our robust results: termite embryos are fungistatic. Our experimental design, robust sample size and statistically significant results stand on their own as novel contributions. We fully agree that protein identification should be the next step; however, it is beyond the scope of this particular manuscript."

4. The authors refer to vitellogenin as a potential (but unlikely) candidate for mediating intra-embryonic antifungal activity (e.g. lines 335-345). Although the molecular weight of vitellogenins can be variable, they are fairly large molecules. However, Figure 1 indicates that the most dominant bands in embryo extracts were < 100 kDa. What is the molecular weight of the subunits of vitellogenin in the species? Did the authors run a SDS-PAGE with egg homogenates as a positive control to identify a similar corresponding protein band? Were there any other assays to establish vitellogenin identity?

We stated in lines 29-30 and 268-269 of the original ms that they were putative vitellogenins, indicating that we were speculating on the identity of those three bands. However, as stated above, we agree that SDS-PAGE gel does not add useful information to the manuscript. Instead, it represents preliminary data in future work to investigate the proteins involved. For this reason, we have removed the SDS-PAGE figure from the results section and revised the ms accordingly.

5. Overall, the results of the SDS-PAGE analysis of embryonic extracts are short and uninformative. For example, it is not clear how the authors infer that "protein profiles validated" and "accurately reflected physiologically distinct embryonic stages". These results need further elaboration, which criteria were used to infer differences? There is no validation or independent characterization of the identity or function of the constituent protein bands. Without these, there is very little inference that can be drawn from the single SDS PAGE.

As state above, we agree with your constructive criticism and have now removed the SDS-PAGE figure from the results section.

6. The result that intraembryonic extracts have more potent antifungal activity than extraembryonic washes needs to be discussed in detail. Do the authors hypothesize that the embryo needing stronger intra-embryonic protection against fungi than external defenses? The authors state that Metarhizium binds and germinates on the outer surface of embryos. If this is true and if the fungus is originating from the soil or is present in the embryonic vicinity, how is the weak antifungal activity of embryonic washes explained. The implications of these results are not clear currently. Is there any information if Metarhizum invades the embryonic chorion or the serosa? If not, what is the justification for testing antifungal activity of intraembryonic contents? The implications and possible causes for a strong intraembryonic antifungal defense need to be discussed further.

As we initiated the research, there was not an *a priori* expectation of whether the outer surface of the egg or the internal contents within the chorion would be more or less fungistatic. As the referee states, natural selection could have selected for different stable strategies. On the one hand, eggs could have been selected to have higher fungistasis on the outer surface of the chorion as a first line of defense. This strategy would ultimately benefit the developing embryo by allowing it to cope with the initial stages of conidia invasion and, thus, mycosis. Alternatively, natural selection could have fostered significant investment in antifungal properties within the chorion at the expense of external protection. This scenario is particularly plausible when considering the social nature of these insects and the intense egg-licking behavior of parents and workers. Parental grooming of eggs could have "emancipated" the developing embryo from investing in external protection and instead invest in within-chorion immune defenses as a secondary layer of protection. Given the lack of comparative data on whether eggs of other social insects exhibit fungistasis, either outside or inside of the chorion, we had no expectations one way or another. Based on previous insect research, we anticipated termite eggs having internal mechanisms to cope with pathogenic burden. First, the yolk protein vitellogenin is known to exhibit antifungal activity (e.g., Salmela and Sunderström, 2017; Sun and Zhang, 2015). Second, some insect eggs have active immune systems (e.g., Jacobs and van der Zee, 2013).

Our aim was modest, using an eco-immunology approach to test the hypothesis that termite eggs exhibit antifungal properties, given that entomopathogenic fungi pose significant selection pressures on other developmental stages of colonies. Our experimental design set out to test this novel hypothesis. Our results indicate that both the outer surface of the chorion and intra-chorionic homogenates have antifungal properties. The ecological and evolutionary implications of our results rest on the recognition that fungal pathogens pose selection pressures, even on the most immature of stages within a termite colony. Evolutionarily speaking, intense brood care (i.e., smearing surface of the chorion with antifungal salivary secretions and the removal of conidia adhering to the outer surface of the chorion) represents a beneficial behavioral response which externally reduces fungal viability and conidia loads. Internally, termite embryos may have also been selected to enhance their immunological responses as they

aged. There is no reason why behavioral (i.e., outer surface of the embryo) and immunological (inside the chorion) adaptations should be pitted against each other. We do not see behavioral and immunological responses as either/or alternatives against disease, but rather complementary to each other.

We have now included some of these explanations into the discussion section in an attempt to provide the reader with more eco-immunological insights regarding the implications and possible causes for embryonic fungistasis in termites.

Appendix B

Reviewer Feedback: Minor Revisions

Author responses in Purple

Dear Ms Cole:

On behalf of the Editors, I am pleased to inform you that your Manuscript RSOS-191418.R1 entitled "Young but not defenseless: Antifungal activity during embryonic development of a social insect" has been accepted for publication in Royal Society Open Science subject to minor revision in accordance with the referee suggestions. Please find the referees' comments at the end of this email.

The reviewers and Subject Editor have recommended publication, but also suggest some minor revisions to your manuscript. Therefore, I invite you to respond to the comments and revise your manuscript.

We thank the editors and reviewers for their review, feedback and decision. Author responses are in purple. Any new revisions have been highlighted in yellow in the revised ms to assist in the subsequent review.

• Ethics statement
If your study uses humans or animals please include details of the ethical approval received, including the name of the committee that granted approval. For human studies please also detail whether informed consent was obtained. For field studies on animals please include details of all permissions, licenses and/or approvals granted to carry out the fieldwork.

We have now added an ethics statement with the permit number for collecting and housing termites at our institution. The sentence "No ethics review or animal care protocol was required for termite research by the USDA or Northeastern University" was also included. Complete statement starts on line **395**.

• Data accessibility
It is a condition of publication that all supporting data are made available either as supplementary information or preferably in a suitable permanent repository. The data accessibility section should state where the article's supporting data can be accessed. This section should also include details, where possible of where to access other relevant research materials such as statistical tools, protocols, software etc can be accessed. If the data has been deposited in an external repository this section should list the database, accession number and link to the DOI for all data from the article that has been made publicly available. Data sets that have been deposited in an external repository and have a DOI should also be appropriately cited in the manuscript and included in the reference list.

If you wish to submit your supporting data or code to Dryad (http://datadryad.org/), or modify your current submission to dryad, please use the following link:
http://datadryad.org/submit?journalID=RSOS&manu=RSOS-191418.R1

Requested statement begins on line **401** of this latest revision.

• Competing interests

Please declare any financial or non-financial competing interests, or state that you have no competing interests.
Statement now begins on line **409**.

• Authors' contributions
All submissions, other than those with a single author, must include an Authors' Contributions section which individually lists the specific contribution of each author. The list of Authors should meet all of the following criteria; 1) substantial contributions to conception and design, or acquisition of data, or analysis and interpretation of data; 2) drafting the article or revising it critically for important intellectual content; and 3) final approval of the version to be published.

All contributors who do not meet all of these criteria should be included in the acknowledgements.

We suggest the following format:
AB carried out the molecular lab work, participated in data analysis, carried out sequence alignments, participated in the design of the study and drafted the manuscript; CD carried out the statistical analyses; EF collected field data; GH conceived of the study, designed the study, coordinated the study and helped draft the manuscript. All authors gave final approval for publication.

Statement begins on line **411**, with "All authors gave final approval for publication" at the end of the statement.

• Acknowledgements
Please acknowledge anyone who contributed to the study but did not meet the authorship criteria.

Statement begins on line **417**.

• Funding statement
Please list the source of funding for each author.

Begins on line **427** – this is a complete list of the funding for this publication.

Please note that we cannot publish your manuscript without these end statements included. We have included a screenshot example of the end statements for reference. If you feel that a given heading is not relevant to your paper, please nevertheless include the heading and explicitly state that it is not relevant to your work.

Thank you for this clarification. We have now amended our ms to include all headings.

Associate Editor Comments to Author:
The reviewers have a number of minor changes for you to tackle, but these should be relatively straightforward to implement.

We thank the Associate Editor for his/her feedback and suggestions, which have surely improved the clarity and overall quality of our work.

Reviewer comments to Author:

Reviewer: 2

Comments to the Author(s)
Thank you for the detailed responses to all my comments.

We thank Reviewer 2 for his/her feedback. We are pleased you are satisfied with our previous responses.

Reviewer: 1

Comments to the Author(s)
First of all I would like to apologize for the delay I have caused in the reviewing process, but I had only sporadic internet access since I was moving in another city.
I thank the authors for clarifying the aspects of the manuscripts that were difficult for me to understand. I only have a few additional comments left.

We thank Reviewer 1 for his/her feedback. These are chaotic times, we hope the move went well :) We have provided additional comments below.

In the statistics section : it would be useful if the authors were stating exactly which model was run for the Kruskal Wallis test, similarly to what they did for the GLMM. If I understood correctly this was germination rate as a response variable as a function of presence of intact or boiled embryonic extract (three levels in the treatment variable : control, unboiled and boiled right ?).

You are correct that the germination rate was the response variable. To perform the KW test, embryonic stage and boiled/unboiled were combined to form a single “treatment” category. Hence, there were 7 levels of the explanatory/independent variable:

1. Control (no embryo homogenate, just fungal conidia)
2. Stage 1 boiled
3. Stage 1 unboiled
4. Stage 2 boiled
5. Stage 2 unboiled
6. Stage 3 boiled
7. Stage 3 unboiled

By combining boiling treatment and egg stage into a new categorical treatment, we were able to compare each of these combinations against the control treatment. This then allowed us to answer the main questions of our research: are different embryonic stages exhibiting different levels of fungistasis and are the compounds responsible for these fungistatic properties heat-labile (pointing toward their degradation, or loss of function). The combined new treatment (boiling treatment + egg stage) also allowed us to compare boiled vs. unboiled for each egg stage when performing the post-hoc Mann-Whitney U tests.

Line **236 – 239** of the revised ms now reads, “Given that the extra- and intra-chorionic protocols differed from one another, we ran separate Kruskal Wallis (KW) tests for each experiment, with germination rate as the response variable. The explanatory variable had 7 levels: controls (fungal conidia only), E1 boiled and unboiled, E2 boiled and unboiled, and E3 boiled and unboiled.”

For the GLMM, the treatment is actually a concatenation of two variables : the embryonic stage and whether the samples were boiled or not. But since the KW test revealed no differences in the germination rates of conidia incubated with boiled samples, these boiled samples can be seen as a control treatment. So then why not test for the interaction between embryonic stage and boiling/not boiling of the samples instead ?
This would make the reading of the results easier, since I suppose for example in Experiment 1 that E1 extra chorionic extracts have a fungistatic activity on conidia compared to E2, E3, and boiled extracts. E2 and E3 on the other hand should not reduce conidia viability compared to boiled extracts.

The reviewer is correct in that the boiled samples are a reasonable substitution for the control treatment, given that the KW test showed no difference between boiled and control samples, irrespective of embryonic stage.

However, we respectfully disagree about testing for an interaction between embryonic stage and boiling. An interaction between 2 variables suggests that the slope of the response variable is different for each permutation of the two variables. However, all boiled samples showed no differences from controls, and, hence, all eggs (regardless of their stage) exhibit the exact same heat-induced effect. In other words, there is a clear main effect of boiling vs. non-boiled that excludes the possibility for an interaction. The two other options for the construction of the model would were 1) Treatment representing embryonic stage only (3 levels: E1, E2 and E3) with boiled/unboiled as a separate explanatory variable, OR 2) as we have presented in the ms, the creation of one combined embryonic stage/boiling treatment variable. While the former would be easier to interpret, the latter allows us to compare the unboiled and boiled groups within the same embryonic stage. **The model currently employed uses each embryonic stage as its own control.** We feel that this allows for a more nuanced interpretation of the results.

Line 283 is confusing : GLMM yielded similar results : the GLMM did not compare the embryonic washes/contents to control, and therefore cannot yield the same conclusion. My comment above might help clarify this.
I think instead of lines 283 to 289, the correct formulation would be “Embryonic stage has a significant effect on the viability of conidia incubated with extra-chorionic washes. Conidia incubated with E1 stage extra chorionic washes have a lower germination rate compared to conidia incubated with E2 and E3 stages (F = 2.1, df = 11, 13488, p =0.06, GLMM).”

Agreed, this was a poor choice of words. The **lines (284)** now read, “After controlling for both COO (Z = 1.1, df = 13488, p = 0.3) and total protein concentration (F = 0.3, df = 1, 13488, p = 0.6), extra-chorionic washes had a marginally significant fungistatic effect on *Metarhizium* conidia (F = 2.1, df = 11, 13488, p =0.06, GLMM). E1 extra-chorionic washes significantly decreased conidia viability relative to all other stages. The antifungal properties of E2 and E3 washes did not significantly differ from each other.”

Lines 302 to 305 : the discrepancy between the results of the model and the results of the post hoc does not indicate anything about the amount of variance explained by the variable. I would instead describe the interaction, and then say that these differences do not hold after Bonferroni correction. Corrections for multiple comparisons are useful when analyzes do indeed require a lot of pairwise comparisons which increases the rate of false discovery, however they have often been criticized for

randomly increasing p-values without regard for effect size. This might be the reason why the interaction does not show during the post hoc.

We agree with the Reviewer's point. However, after we investigated this interaction further by creating scatter plots depicting Germination rate as a function of Total protein, it was evident that the differences in slopes for each graph were driven by outliers in total protein, and the R^2values remain small. These figures, in combination with the post-hoc comparisons for the interaction Total Protein*Treatment, indicate that total protein does not explain differences in germination rates. We have now added a new Supplemental Figure 2 to address this concern and altered the results section to read:

Line 302 "Although the interaction treatment × total protein was significant (F = 2.7, df = 5, 14288, p = 0.02), these significance appears to have been driven by the total protein outliers; none of the post-hoc comparisons were significantly different from each other. Graphs of these interactions also support the conclusion that total protein explains only a small amount of the variation observed between treatments (with R^2 estimates ranging from 0.02 to 0.25; Supplemental Figure 2)."
